# Analysis of Environmental DNA and Edaphic Factors for the Detection of the Snail Intermediate Host *Oncomelania hupensis quadrasi*

**DOI:** 10.3390/pathogens8040160

**Published:** 2019-09-23

**Authors:** Fritz Ivy C. Calata, Camille Z. Caranguian, Jillian Ela M. Mendoza, Raffy Jay C. Fornillos, Ian Kim B. Tabios, Ian Kendrich C. Fontanilla, Lydia R. Leonardo, Louie S. Sunico, Satoru Kawai, Yuichi Chigusa, Mihoko Kikuchi, Megumi Sato, Toshifumi Minamoto, Zenaida G. Baoanan, Marcello Otake Sato

**Affiliations:** 1 Department of Biology, College of Science, University of the Philippines Baguio, Governor Pack Road, Baguio City 2600, Philippines; fccalata@up.edu.ph (F.I.C.C.); caranguiancamille@gmail.com (C.Z.C.); jmmendoza8@up.edu.ph (J.E.M.M.); zgbaoanan@up.edu.ph (Z.G.B.); 2 DNA Barcoding Laboratory, College of Science, National Science Complex, University of the Philippines Diliman, Quezon City 1101, Philippines; lydialeonardo1152@gmail.com; 3 Natural Sciences Research Institute, College of Science, National Science Complex, University of the Philippines Diliman, Quezon City 1101, Philippines; 4 College of Medicine, University of the Philippines Manila, Pedro Gil St. Ermita, Manila 1000, Philippines; iankimbasastabios@gmail.com; 5 Graduate School, University of the East Ramon Magsaysay Memorial Medical Center, 64 Aurora Blvd., Quezon City 1100, Philippines; 6 Rural Health Unit, Municipal Health Office, Gonzaga, Cagayan Valley 3515, Philippines; gacuscusmark@gmail.com; 7 Department of Tropical Medicine and Parasitology, Dokkyo Medical University, 880 Kitakobayashi, Mibu-machi, Shimotsuga-gun, Tochigi 321-0293, Japan; skawai@dokkyomed.ac.jp (S.K.); ychigusa@dokkyomed.ac.jp (Y.C.); 8 Department of Immunogenetics, Institute of Tropical Medicine, Nagasaki University, 1-12-4 Sakamoto, Nagasaki 852-8523, Japan; mkikuchi@nagasaki-u.ac.jp; 9 Graduate School of Health Sciences, Niigata University 2-746 Asahimachi-dori, Chuo-ku, Niigata 951-8518, Japan; satomeg@clg.niigata-u.ac.jp; 10 Graduate School of Human Development and Environment, Kobe University, 3-11, Tsurukabuto, Nada-ku, Kobe 657-8501, Japan; minamoto@people.kobe-u.ac.jp

**Keywords:** *Oncomelania hupensis quadrasi*, schistosomiasis japonica, environmental DNA, edaphic factors, snail surveillance

## Abstract

Background: The perpetuation of schistosomiasis japonica in the Philippines depends to a major extent on the persistence of its intermediate host *Oncomelania hupensis quadrasi,* an amphibious snail. While the malacological survey remains the method of choice in determining the contamination of the environment as evidenced by snails infected with schistosome larval stages, an emerging technology known as environmental DNA (eDNA) detection provides an alternative method. Previous reports showed that *O. hupensis quadrasi* eDNA could be detected in water, but no reports have been made on its detection in soil. Methods: This study, thus focused on the detection of *O. hupensis quadrasi* eDNA from soil samples collected from two selected schistosomiasis-endemic barangays in Gonzaga, Cagayan Valley using conventional and TaqMan-quantitative (qPCR) PCRs. Results: The results show that qPCR could better detect *O. hupensis quadrasi* eDNA in soil than the conventional method. In determining the possible distribution range of the snail, basic edaphic factors were measured and correlated with the presence of eDNA. The eDNA detection probability increases as the pH, phosphorous, zinc, copper, and potassium content increases, possibly indicating the conditions in the environment that favor the presence of the snails. A map was generated to show the probable extent of the distribution of the snails away from the body of the freshwater. Conclusion: The information generated from this study could be used to determine snail habitats that could be possible hotspots of transmission and should, therefore, be targeted for snail control or be fenced off from human and animal contact or from the contamination of feces by being a dumping site for domestic wastes.

## 1. Introduction

Schistosomiasis, a snail-borne parasitic infection, is one of the most important neglected tropical diseases (NTDs) that continues to prevail and remains a significant public health problem in 76 tropical and subtropical countries [1,2,3,4]. The persistence of schistosomes depends highly on the continued presence of certain species of snails that serve as intermediate hosts [5,6,7,8]. There are seven major species of schistosomes that infect man but one species in particular, *Schistosoma japonicum*, is the most virulent and is considered a true zoonosis [7,9,10,11,12,13,14]. Mammals like cattle, water buffalo, goats, cats, dogs, pigs, and rodents serve as reservoir hosts [14,15]. *S. japonicum* infects an unsuspecting host via skin penetration if exposed in cercariae-contaminated freshwaters [5,6,14].

*Oncomelania hupensis* is a gastropod species serving as the intermediate host of *S. japonicum* in at least five countries in east and southeast Asia including the Philippines [8,16]. Currently, there are nine recognized subspecies of *O. hupensis* with taxonomic subspecies classification assigned according to their region of endemicity [16]. Four subspecies occur in China, two in Taiwan, and a single subspecies is observed each in Japan, the Philippines (*O. hupensis quadrasi*), and Indonesia [16,17,18,19]. However, recent studies based on molecular data refute most of the subspecies designation based on the geographical distribution [19]. 

Among the nine *O. hupensis* subspecies, *O. hupensis quadrasi* is the most amphibious [16]. It is capable of thriving both in aquatic freshwater environments as well as in muddy to dry soil areas. Under prolonged conditions of drought, *O. hupensis quadrasi* closes its operculum, burrows into the soil, and aestivates until such time when moisture returns through rain or floods [20]. This snail can thrive in both natural and man-made freshwater habitats and could be dispersed through flooding. Snail colonies in established sites are waterlogged and well-shaded [21], and their distribution in an area was reported to be influenced by breeding and survival through the variation of the soil pH and calcium [22]. Thick vegetation that provides shade and anchorage creates a suitable microclimate for the snail to thrive and reproduce [16,23,24]. 

**Figure 1:**
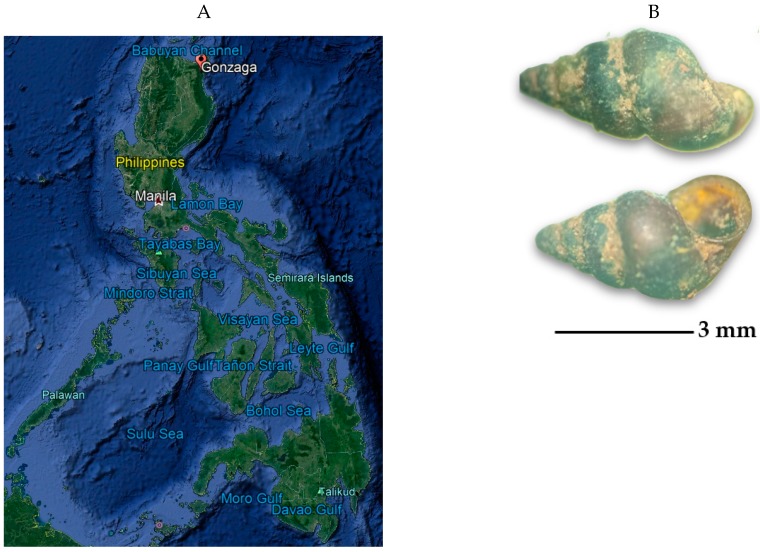
(**A**) Map showing Gonzaga, Cagayan Valley in the Philippines [32]; (**B**) *O. hupensis quadrasi*, the snail intermediate host of *S. japonicum* in the Philippines, collected from Gonzaga, Cagayan Valley. The upper panel shows the aboral side and the lower panel shows the oral side where the snail’s operculum could be seen.

Endemicity of schistosomiasis used to be observed only in provinces experiencing wet season all throughout the year until the discovery of new endemic foci in 2002 in Gonzaga, Cagayan Valley located up north (Figure 1A), an area known for summer temperatures reaching as high as 40 °C and extremely dry conditions. This discovery shows a potential adaptation of the snail to such conditions [20,25]. 

Schistosomiasis control in the Philippines has a long history dating as far back as 1951 when the Division of Schistosomiasis was created by the Ministry of Health [8,9,12]. Control of *O. hupensis quadrasi* snails (Figure 1B) was mainly through environmental modification, such as filling up of waterlogged areas and cementing dikes and canals or through chemical mollusciciding, which were all difficult to sustain because of the high cost and the huge demand for manpower, not to mention the very extensive areas of potential snail habitats to cover [20,26]. The shift from snail control to morbidity control was made in the early 1980s with the discovery of a cheap and effective drug, praziquantel, for all forms of schistosomiasis. Since then, mass drug administration using praziquantel has been the cornerstone of schistosomiasis control and prevention by the Philippine Department of Health (DOH). However, schistosomiasis in the Philippines is still endemic in 12 regions, 28 provinces, 14 cities, 203 municipalities, and 1,593 barangays (villages) wherein 12.4 million Filipinos are at risk of infection, and 3.4 million are directly exposed [8]. 

Schistosomiasis surveillance is performed to monitor progress of control programs, especially when prevalence levels have gone down to elimination levels. One specific conventional surveillance technique is the malacological survey where *O. hupensis quadrasi* snails are collected, crushed, and examined for the presence of the characteristic furcocercous cercariae of schistosomes [8,27]. The presence of infected snails is an indication of environmental contamination by fecal matter containing schistosome eggs in the freshwater [7]. Though this method is cost-effective, the demand for expertise in correctly identifying the snails in their natural habitat and for the mobilization of huge manpower during surveys, and the inaccessibility of some snail sites make the malacological survey a huge task to undertake [8,26,27]. 

The use of environmental DNA (eDNA) has been demonstrated to be useful in tracking *S. japonicum* in water through quantitative PCR (qPCR) [28,29]. It was also successfully applied to other schistosome species, such as *S. mansoni* in field samples for better surveillance [30,31]. In this study, *O. hupensis quadrasi* eDNA was detected from soil samples. The use of this type of DNA source material utilizing soil samples could be a useful tool for snail surveillance due to the amphibious nature of the snail.

In this study, detection of *O. hupensis quadrasi* was performed using the eDNA detection technique from soil samples collected from Gonzaga, Cagayan Valley (Figure 1A). eDNA detection was compared from snail sites harboring *O. hupensis quadrasi* and adjacent areas where no snails were observed through the classical malacological survey. Complementary, edaphic factors with putative influence in the distribution of *O. hupensis quadrasi* were investigated. 

## 2. Results

### 2.1. Description of Collection Sites and Malacological Survey

**Figure 2:**
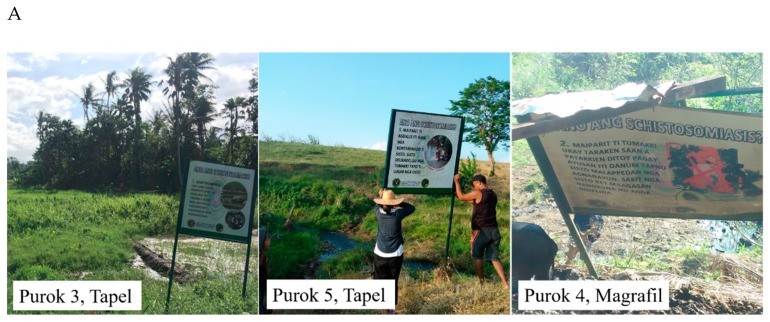

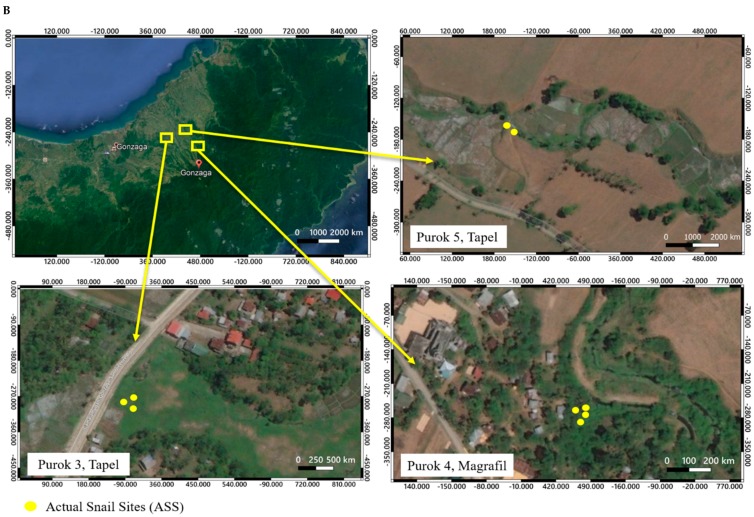

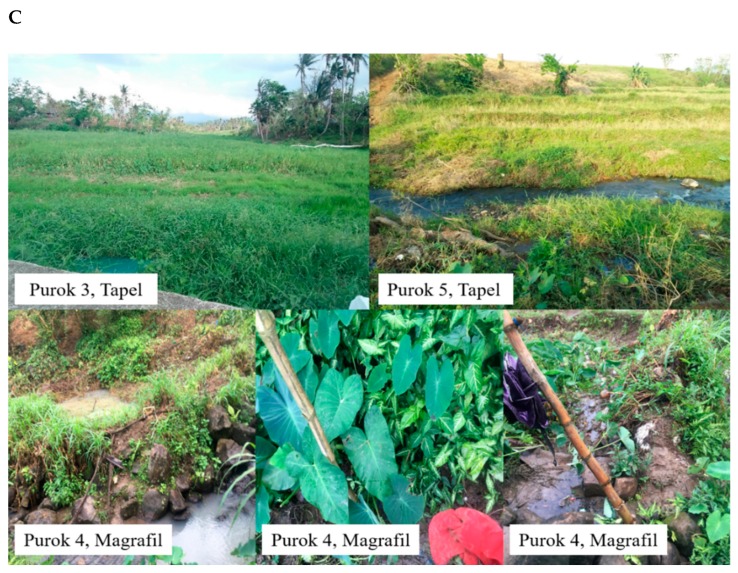
(**A**) Signages of schistosomiasis-endemic sites in Gonzaga, Cagayan Valley; (**B**) Actual snail sites mapped using qGIS 3.6.0. visited in barangays Tapel and Magrafil for soil collection; (**C**) Sampling collection sites in Purok 3, Tapel, Purok 5, Tapel, and Purok 4, Magrafil.

The collection sites were initially determined by the presence of signages indicating infested areas (Figure 2A). A total of 27 sampling points were established: 9 from Purok 3 and 6 from Purok 5, Tapel, and 12 from Purok 4, Magrafil (Figure 2B). The sampling points positive for live *O. hupensis quadrasi* based on the malacological survey were indicated as Actual Snail Site (ASS) while two other sampling points 1 meter away from ASS were designated as Potential Snail Site (PSS). The actual snail sites were located in the map using Quantum Geographic Information System (qGIS v3.6.0) (Figure 2B). Please refer to the section on Materials and Methods for details.

The sampling areas are shown in Figure 2C. Purok 3, Tapel was a flat grassy field with swampy parts and was adjacent to a rice field. *O. hupensis quadrasi* populations were found to be randomly distributed in small rocks and on the stems of grasses in the swampy areas. The waterlogged and thick vegetation provided enough moisture and shade for the snails to establish a stable colony in the area. The snails in Purok 5, Tapel were also distributed in patches along the margins of the stream. These margins were often used as cooling and watering holes for carabaos. Farmers, along with their carabaos and pet dogs, would cross the stream on their way to their rice fields and corn plantations. Snails observed in Purok 4, Magrafil occurred in a clumped distribution and thrived along margins of a shallow stream with shady areas planted with Gabi (*Colocasia esculenta*) and Palauan (*Cyrtosperma merkusii*) while small ponds were likewise observed to support colonies of *O. hupensis quadrasi*. The collection area was contaminated with litter and fecal matter from livestock. Moreover, there were residents who bathed and washed clothes in the stream. 

### 2.2. eDNA Detection via Conventional PCR and qPCR

All the 81 eDNA samples were run in triplicates using both conventional PCR and qPCR. (Supplementary Material Table S1). Conventional PCR detected *O. hupensis quadrasi* eDNA in only one of the 27 sampling points (data not shown). On the other hand, qPCR detected *O. hupensis quadrasi* eDNA in 22 out of 27 (81.48%) sampling points as indicated in the Ct values and amplification plots (Supplementary Material Table S2). 

**Figure 3:**
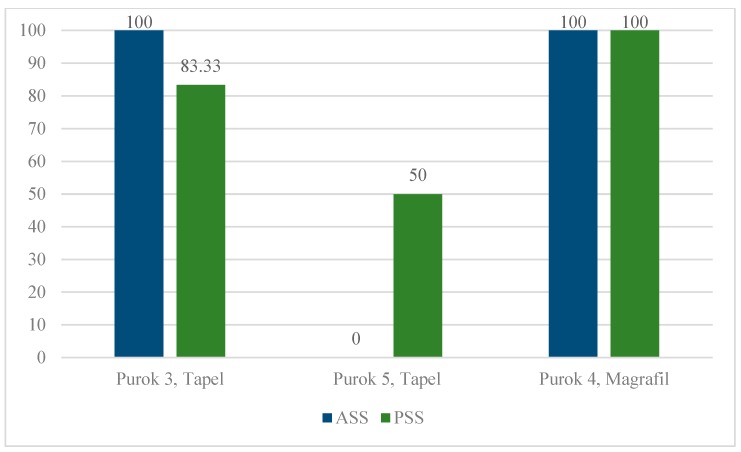
eDNA detection rate of *O. hupensis quadrasi* from actual snail sites (ASS) and potential snail sites (PSS) using qPCR in three Puroks from barangays Tapel and Magrafil, Gonzaga, Cagayan Valley.

Specifics of eDNA detection rate is summarized in Figure 3. In Purok 3, Tapel, all ASS (100%) had detectable eDNA, whereas all 5 out of 6 PSS were positive for eDNA (83.33%). In Purok 5, Tapel, no eDNA was detected in 2 ASS visited, whereas 2 out of 4 PSS (50%) had detectable eDNA. Moreover, eDNA was detected in all sampling areas in Purok 4, Magrafil.

## 3. Distribution Mapping of eDNA Positive Sites

**Figure 4:**
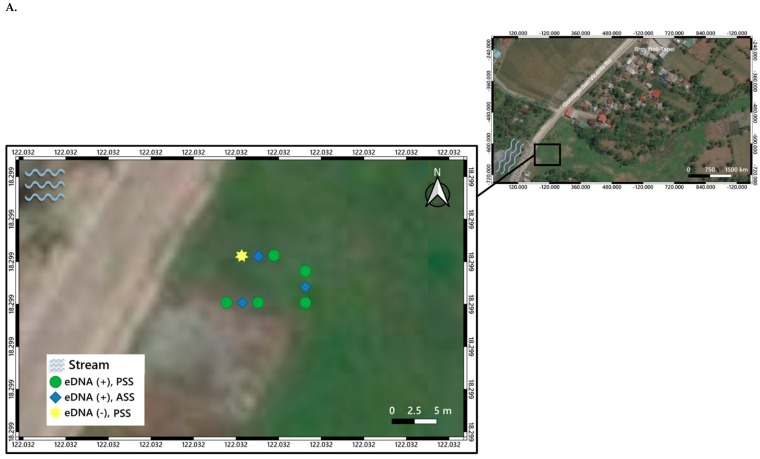

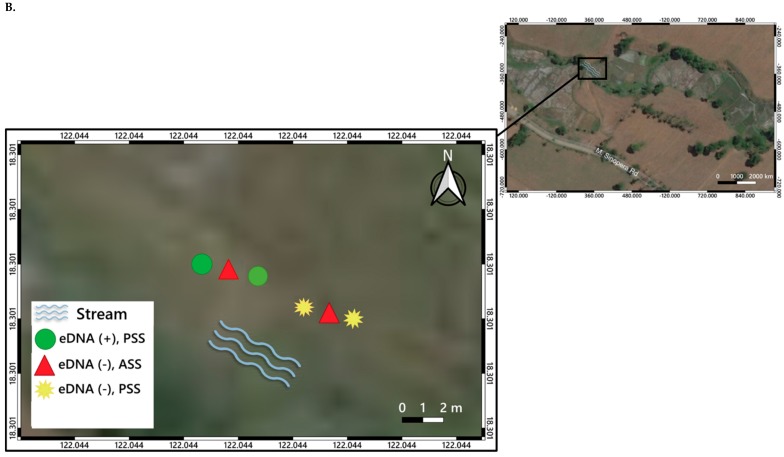

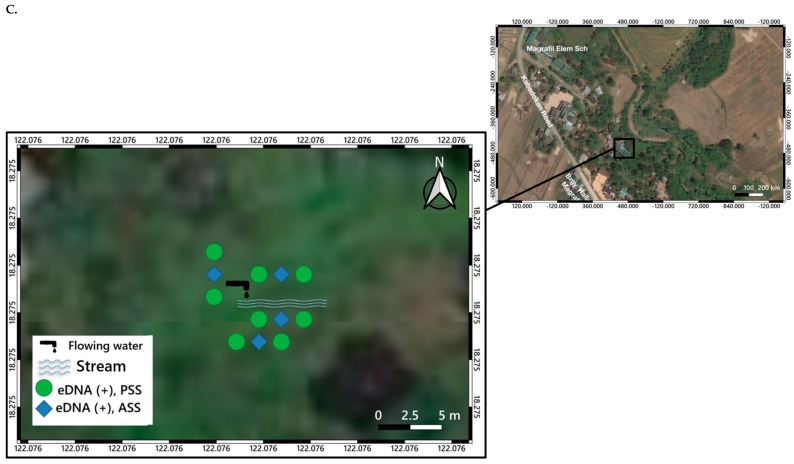
Actual sampling areas of ASS and PSS plotted in qGIS v.3.6.0. using recorded Global Positioning System (GPS) points either positive or negative to *O. hupensis quadrasi* eDNA of sampled areas of (**A**) Purok 3, Tapel, (**B**) Purok 5, Tapel, and (**C**) Purok 4, Magrafil.

Specific location points of ASS and PSS classified according to the results of qPCR detection of *O. hupensis quadrasi* eDNA were determined using Global Positioning System (GPS) and plotted in the map using qGIS (Figure 4A–C). Water bodies such as streams as observed during the field visit were also incorporated in the map as indicated in the legend of each map generated.

## 4. Soil Edaphic Factors 

**Figure 5:**
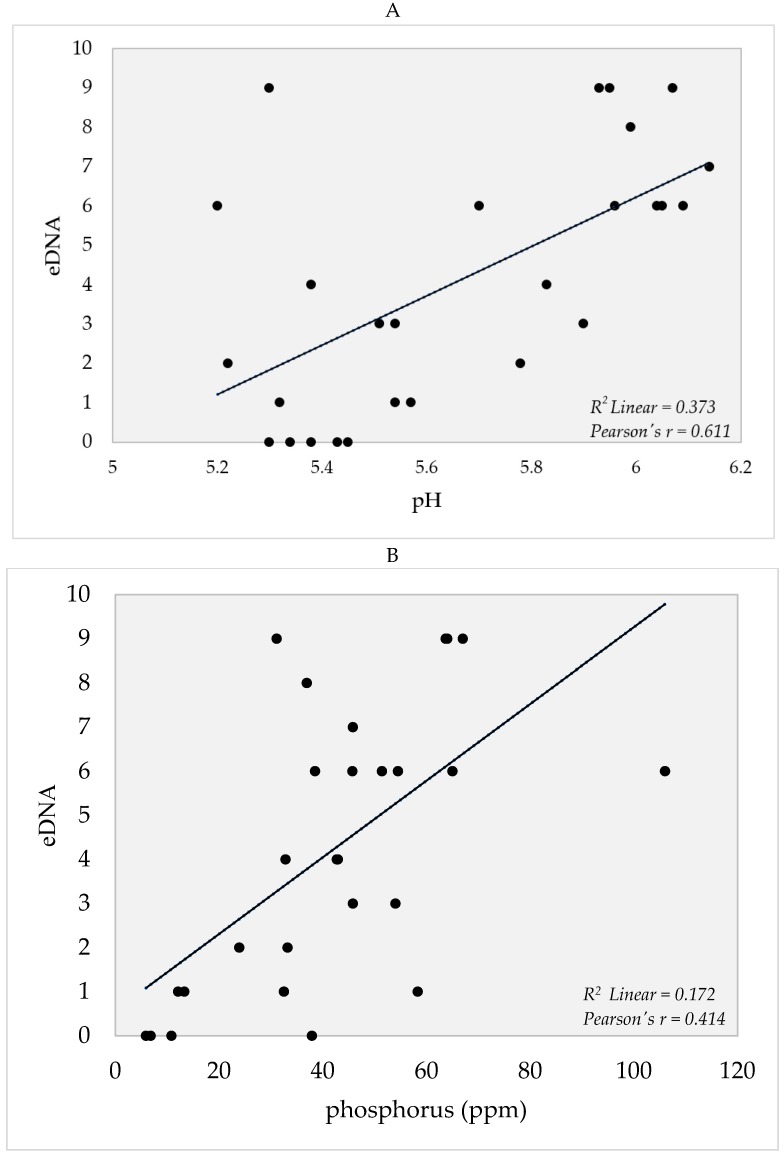

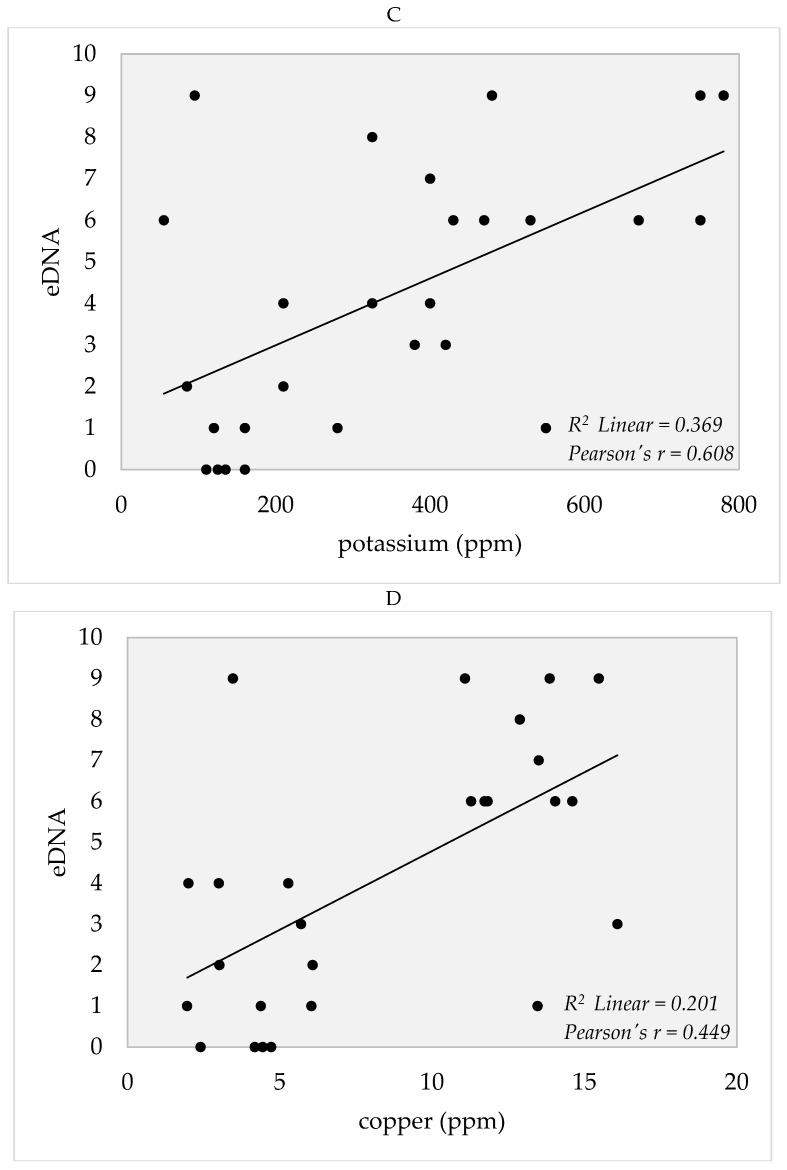

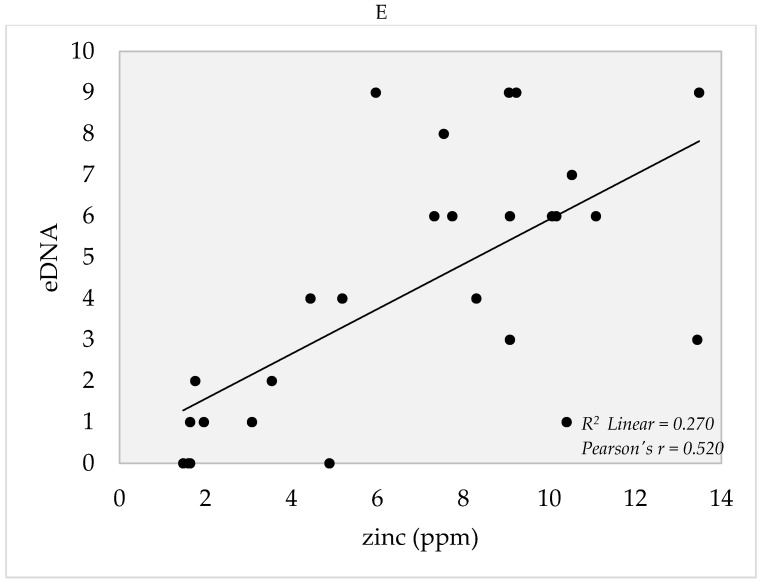
Positive correlation scatter plots of eDNA detection probability vs. (**A**) pH, (**B**) phosphorus, (**C**) potassium, (**D**) copper, (**E**) zinc calculated using Pearson Correlation in SPSS v. 16.

Edaphic factors that were measured are summarized in Supplementary Material Table S3. These factors were correlated to the eDNA detection probability (number of positive eDNA readings in the qPCR of a sample) using Statistical Package for the Social Sciences (SPSS). There were positive correlations observed between edaphic factors, such as pH, P, K, Cu, and Zn (Figure 5A–E) in which higher detection rates were observed in samples with increasing levels of the aforementioned factors in the soil. On the other hand, no correlations were observed for temperature, Ca, Mn, N, Fe, and organic matter (data not shown). 

## 5. Discussion

### 5.1. Detection of eDNA of O. hupensis quadrasi from Soil Samples via Conventional PCR and qPCR 

Results of eDNA detection using conventional PCR and qPCR are summarized in Supplementary Material Table S1 and Figure S1. eDNA was detected using both conventional PCR and qPCR, but detection rates in the latter were higher. The use of TaqMan-qPCR also addresses the problem of the production of non-specific fluorescence signal from non-specific amplification [27]. This is due to the high-sequence specificity of the primers and probes designed, and a signal is only generated if the binding to the target sequence of the primers and probes to the target DNA is observed in the reaction. The utilization of probes with the primers increases not only the sensitivity of the qPCR but most importantly the specificity of the reaction.

The A260/230 ratios of spectrophotometry of the soil samples confirm the presence of protein contaminants and organic compounds. In the samples, the values were extremely lower than the ideal range, which indicates that the eDNA extracts were not pure and contain significant contaminants such as proteins, carbohydrates and inorganic compounds such as salts that can inhibit PCR. The qPCR negative results observed in ASS in Tapel (Figure 3 and Figure 4A,B) may be due to compounds acting as inhibitors during PCR which results in zero amplification even if snails are observed in these sites [33,34]. Another plausible reason could be the rapid degradation of DNA before it even settled to the sediment. It is possible to detect snails in the field, but little to no eDNA could be detected in soil sediments due to the rapid degradation processes to which unbound DNA are exposed to [35,36]. The amount of eDNA that has settled on the soil sediment is dependent on the rate of eDNA shedding of the source organism. Failure to collect the soil with the target DNA even though live *O. hupensis quadrasi* is present at the site is therefore possible. To address this problem, extensive sampling points were designed to systematically collect soil from these areas so that the probability of detecting eDNA would be higher. Nevertheless, the detectable eDNA of *O. hupensis quadrasi* confirmed the efficacy of eDNA detection through qPCR even with high carbohydrate carryover and low initial DNA concentration. As the probability of detecting eDNA and the ability to recover it varies with eDNA concentration, the inconsistencies of the detection among qPCR replicates (Supplementary Material Table S1) are anticipated since the concentration of the initial target DNA is extremely low [36,37]. At least one positive result from the three repeated runs for a particular sample in the qPCR test was considered positive to *O. hupensis quadrasi* eDNA.

The utilization of eDNA detection will enhance the efficiency of the snail surveillance, most especially in areas where the snail density is low, and the distribution is too patchy. Using soil as material, it is also possible to detect potential snail habitats that may harbor snails even during the dry season. Since the malacological survey as a surveillance technique is done routinely in some endemic areas in the Philippines, eDNA detection could be applied as a complementary technique to confirm the presence of the snails or if the area has been inhabited by the snail since eDNA could persist for a long duration of time. Although the current study did not include the detection of *S. japonicum* using water, it is more relevant to track the parasite using water due to its high dependability on the medium to complete its life cycle [7,8,27]. Nevertheless, detection of *S. japonicum* using soil samples could also be explored since it may indicate the presence of infected snails. It can also determine if the sampled area is a site of active transmission where continuous environmental contamination is experienced through the presence of miracidia emerging from eggs introduced through the fecal matter from infected hosts to freshwater bodies. The detection of *S. japonicum* in soil samples may also provide useful geographic implications on the range of cercarial displacement in dry areas where flooding is prominent. This could also be achieved by using *S. japonicum* primers and *S. japonicum* specific probes to increase the sensitivity and specificity of the detection using qPCR. However, since *S. japonicum* is not visible to the naked eye, it would be difficult to assign sampling points for detection.

Although eDNA detection can provide an alternative method to survey snails, this method cannot assess the typical density of *O. hupensis quadrasi* and its real-time location, whether it comes from a recently sloughed-off DNA or a dead snail [38,39,40,41]. The success of using the eDNA method further depends on certain characteristics of the species of interest. For example, it is easier to detect species such as fish and amphibians with slimy skin since they release great quantities of DNA in the environment as compared to arthropods which only release small amounts [39]. In terms of *O. hupensis quadrasi*, its high dependability and contact with soil and water increase the chance of eDNA shedding, making either water or soil a good candidate material to use to detect them.

### 5.2. O. hupensis quadrasi eDNA Potential Distribution

In Purok 3, Tapel, all ASS and 5 PSS were positive for eDNA (Figure 4A), which indicates that even if there was absence of snails in the PSS during sampling, it could mean that *O. hupensis quadrasi* could have been in the site in the past or its eDNA could have been transported to nearby PSS. The presence of eDNA in some PSS may imply that the snails were present in the site but were missed during the malacological survey or that the eDNA could have originated from a nearby ASS as eDNA exhibits high mobility. 

No eDNA was detected in all ASS and two PSS were positive with eDNA in Purok 5, Tapel (Figure 4B). The presence of *O. hupensis quadrasi* eDNA in PSS indicates a potential snail habitat. Moreover, the presence of eDNA indicates the possible distance of eDNA dispersion from the nearest source such as an actual snail site, which was used as a reference during sampling. *O. hupensis quadrasi* spawn and sometimes shed *S. japonicum* cercariae along the stream, then return to the soil substrate where they settle and possibly aestivate. In Purok 4, Magrafil, all ASS and PSS were positive for eDNA (Figure 3; Figure 4C), which may be attributed to the high abundance of snails. It can be inferred from the maps that the snail can travel 0.5 m to 6.5 m from the stream to the sampling points. In addition, it is possible that *O. hupensis quadrasi* is dispersed by livestock animals, humans, or by continuously flowing water. 

It is difficult to accurately track the source and movement of DNA molecules since eDNA data relies on inference [42]. For instance, dispersion and dilution of eDNA may be affected by stream currents and wave actions [39,43]. In the case of *O. hupensis quadrasi*, the presence of eDNA may confirm the extent of snail distribution after flooding. eDNA detection is easier in species living in small isolated areas since sampling could be limited to their particular habitats. Meanwhile, eDNA detection could be difficult in species with greater means of dispersal living in big rivers or terrestrial habitats since sampling would have to be extensive. There is a high probability of positive eDNA detection if the target species is in the area recently since DNA degrades over time [39]. Thus, it is essential to have an appropriate sampling design based on whether the collected sample is near or far from the target species.

### 5.3. Edaphic Factors and eDNA Detection

Results of measured edaphic factors in sampling areas are summarized in Supplementary Material Table S3. There were positive correlations in the eDNA detection probability with the edaphic factors pH, P, K, Zn, and Cu (Figure 5). On the other hand, no correlations were found with temperature, N, Fe, Ca, Mn, organic matter, and organic carbon (data not shown). Knowledge of these parameters, which may regulate the local distribution of *O. hupensis quadrasi* or the persistence of the snail’s DNA, is necessary to determine the parameters’ effects on the distribution of the snail.

There was a moderate positive correlation (ρ = 0.611) in the pH with eDNA detection (Figure 5A). Although pH values increased as the number of eDNA positive readings in qPCR increased, increasing pH may not necessarily mean that *O. hupensis quadrasi* could tolerate a very basic environment since the snail generally thrives at a range of 4.6 to 9 [22]. The positive correlation in our results, however, may imply that *O. hupensis quadrasi* could tolerate slightly acidic environments (5.20 to 6.14) or the snail’s DNA is stable in these pH conditions. If the pH becomes very basic, the *O. hupensis quadrasi* productivity becomes low [22], whereas a very acidic environment promotes DNA degradation and shell erosion, which may lead to death [44]. 

eDNA detection had a moderate positive correlation (ρ = 0.414) with phosphorus (P) (Figure 5B). The P range of 5.9 to 106 ppm may indicate that *O. hupensis quadrasi* can thrive within this range and detection of the snail’s DNA is possible. Pesigan and colleagues [21] found that P concentration has no effect on the distribution of the snails in Palo, Leyte. However, the results of this study point otherwise. This is supported by a study by Johnson and colleagues [45], which found that P has an effect on the snail distribution since P can serve as a nutrient. Increased amounts of P may be due to the sewage released by nearby households in Gonzaga, which allowed the perpetuation of *O. hupensis quadrasi* and the eventual increase of detected eDNA [46]. 

The probability of detecting eDNA was also noted to increase with increasing potassium (K) levels, showing a moderate positive correlation (ρ = 0.608) (Figure 5C). The increasing K levels may be due to the high moisture available in the soil during sampling, which was performed during the wet season. Moisture permits K leaching from crop residues (*Oriza sativa* and *C. esculenta*) to soil [47]. Thus, moisture might still be available to *O. hupensis quadrasi* despite being 0.5 to 6.5 m away from the stream (Figure 5B,C). The positive correlation between K and detected eDNA might, therefore, infer a possible tolerance of *O. hupensis quadrasi* to ecotoxic potassium-based compounds, such as potassium nitrate (KNO_3_) and potassium chloride (KCl) [47]. 

Copper (Cu) also showed a moderate positive correlation (ρ = 0.449) with number of detected eDNA (Figure 5D). The correlation may reveal a possible tolerance of the snail to increasing Cu (up to 16.08 ppm) since this element is toxic to snails with increasing amounts [48]. In other gastropod species such as *Melanoides tuberculata* and *Theodoxus niloticus*, the mean lethal concentrations to Cu are 0.14 ppm and 8.6 ppm, respectively [48,49]. A study by Moreno and McCord [50] showed that high Cu has adverse effects on DNA processing by directly interacting with the DNA, thereby altering its chemical structure [51]. Moreover, Cu-based compounds such as copper sulfate (CuSO_4_), copper pentachlorophenate, and copper-controlled release glass (CRG) are used as molluscicides to intermediate hosts of *Schistosoma* spp. [52,53]. CuSO_4_ specifically is known to be toxic to snails in minute doses for as low as 2 ppm, which can cause death after 48 hours of exposure [52]. 

Zinc (Zn) also showed a moderate positive correlation (ρ = 0.520) (Figure 5E). Since compounds of Zn such as ZnO nanoparticles serve as molluscicides to *Oncomelania* snails and *Biomphalaria alexandrina* [54,55], the positive correlation of zinc with eDNA detection may indicate a possible tolerance of *O. hupensis quadrasi* to rising zinc levels (up to 13.48 ppm; Supplementary Material Table S3). The mean lethal concentrations of 3.9 ppm and 12.2 ppm occur in *M. tuberculata* and *T. niloticus.* There is a high probability that zinc-based consumer products (e.g., batteries) leach out from Zn-contaminated sites to the endemic sites [56].

Temperature, Ca, Mn, N, Fe, organic matter, and organic carbon showed no significant correlation with the eDNA detected in soil. Although *O. hupensis quadrasi* is known to be sensitive to changes in temperature, the increasing temperatures did not affect eDNA detection since sampling was only performed during the wet season and only minute differences were observed in soil temperatures [21,22,57]. Calcium is critical to the survival of *O. hupensis quadrasi* as it is needed for the development of their calcareous shell [58]. Hence, increasing calcium content should result in increasing the probability of detecting the snail’s eDNA. However, results in this study show the absence of a correlation between eDNA detection and calcium. It is hypothesized that the three-month gap between soil collection and soil analysis altered the initial calcium content of the samples. Similarly, a study by Pesigan et al. [21] suggests that calcium have no effect on snail distribution in Palo, Leyte. Nevertheless, based on this study, it is possible that the snail could survive or its eDNA could persist in varying concentrations of calcium (0.25–10 cmol/kg), organic carbon (0.9–3%), and organic matter (1.5–5.3%).

Furthermore, the absence of *O. hupensis quadrasi* in places appearing to be suitable for them led McMullen to conclude that changes in the environment may be responsible for the altered tolerance range of *O. hupensis quadrasi* [21,23]. Results from this study, however, do not indicate the overall general trend for the soil factors affecting *O. hupensis quadrasi*, as other environmental factors may affect the presence of *O. hupensis quadrasi* eDNA. Furthermore, the trend in the correlation of the eDNA detection probability and edaphic factors measured may change seasonally since sampling was only done for one season for this study. More extensive temporal trends in the variables measured may provide higher resolution of eDNA detection probability and edaphic factors that may affect the distribution of *O. hupensis quadrasi* in an endemic area.

## 6. Conclusions and Recommendations

This is a pioneering study showing the success of detecting eDNA of *O. hupensis quadrasi* in soil despite the low initial DNA concentrations of the target organism and the presence of inhibitors. eDNA was detected in 22 out of 27 sites (81.48%) of barangay Tapel and Magrafil via qPCR while only one out of 27 sampling sites (3.70%) tested positive using conventional PCR, validating the sensitivity of qPCR over conventional PCR. Distribution mapping confirmed that *O. hupensis quadrasi* could extend their habitat range or their eDNA could be dispersed from 0.5 m to 6.5 m away from the stream. This could also indicate the ability of the snails to thrive in relatively drier areas which may be far from water sources. Pearson’s positive correlation showed that the detection of eDNA (number of positive readings in qPCR) increases as the pH, phosphorous, potassium, copper and zinc content increase. The correlation may infer a possible tolerance range of *O. hupensis quadrasi* or persistence of its eDNA in these conditions. Thus, the initial snail distribution provides basic information about the snail intermediate host, which could be further analyzed to mitigate the threats of schistosomiasis in endemic areas.

The limitations and benefits of eDNA detection should be evaluated when selecting a method in targeting vectors, intermediate hosts, and parasites. Seasonal changes of eDNA concentrations and biomass or the abundance through eDNA quantification in the soil could be further studied. Detection of eDNA of both the intermediate snail host and the parasite is also suggested to determine the extent of transmission of schistosomiasis in an endemic area. The effect of temperature on eDNA distribution could also be evaluated throughout the year, involving the sampling during wet and dry seasons to determine the possible tolerance range and adaptation of *O. hupensis quadrasi. O. hupensis nosophora* found in Japan, which used to be endemic for schistosomiasis until it was eliminated in the 1990s, experienced a drastic reduction in the wild populations due to intensive snail control measures as part of the intensive campaign to eliminate and permanently interrupt transmission of the disease [17]. The same is observed in some subspecies of *O. hupensis* in China, where intensive mollusciciding and physical environmental modification are still practiced [18].

## 7. Materials and Methods

### 7.1. Soil Sampling, Malacological Survey, and Mapping of Snail Sites

**Figure 6:**
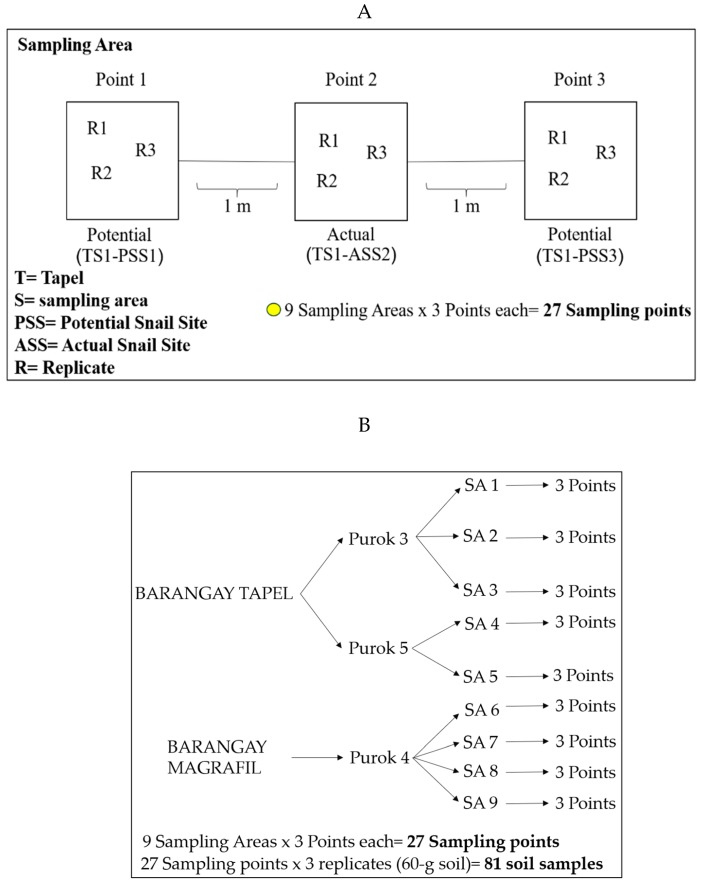
(**A**) Established ASS and PSS in one sampling area; (**B**) Overall sampling points and soil samples in barangay Tapel and Magrafil.

Soil sampling was conducted in January 2019 in schistosomiasis-endemic areas in Purok (zone) 3 and Purok 5 of Barangay (village) Tapel and Purok 4 of Barangay Magrafil in Gonzaga, Cagayan from actual snail sites (ASS) and potential snail sites (PSS). ASS are places with snails confirmed through the intensive malacological survey in which at least one hour was spent searching for snails. The PSS sites were established as two quadrats at one-meter distance to the left and right of ASS with the confirmed absence of snails by the malacological survey (Figure 6A). 

Overall, there were nine sampling areas (Figure 2B and Figure 6A): three in Purok 3, Tapel; two in Purok 5, Tapel; and four in Purok 4, Magrafil, with nine ASS sampling points and 18 PSS sampling points for a total of 27 (Figure 6A,B). In each sampling point, three replicates of soil samples were collected accounting to a total of 81 soil samples (Figure 6B). The GPS coordinates of all sampling points of ASS and PSS, whether positive or negative to *O. hupensis quadrasi* eDNA, were also recorded and mapped (Figure 4). 

To perform the malacological survey, five people were involved. Sites with actual snails were screened by intensively searching for live *O. hupensis quadrasi*. Appropriate protective clothing such as knee-level boots and gloves were used. Snails were collected with the use of forceps and were stored in properly labeled plastic cups. Photographs and GPS coordinates using a portable GPS device (Etrex H, Garmin) were taken and recorded for each of the established snail sites and were mapped using qGIS 3.6.0 (Figure 2B). 

Soil samples were taken at a depth of approximately 10 cm. For each ASS and PSS sites, triplicate soil samples of about 60 g were collected and placed separately in properly labeled polyethylene bags (size 8” × 10”) for eDNA extraction. Another 500 g sample of soil was set aside from each sampling area (Tuguegarao, Cagayan Valley, and Capas, Tarlac) for soil quality analysis. During the soil collection, the trowel used for each site was rinsed with a 10% bleach solution after every sampling to prevent cross-contamination. Shoulder-length gloves were used in soil collection to avoid exposure to the parasite. 

### 7.2. Storage of Soil Samples and Measurement of Edaphic Factors

The soil samples were transported to the Oven Room, KA Building of the University of the Philippines Baguio for further processing. Samples were oven-dried at ~40 °C for about two weeks. Dried soil samples were submitted to the Department of Agriculture Region II and III Soils Laboratory for analyses of soil pH, phosphorus (P), potassium (K), zinc (Zn), copper (Cu), manganese (Mn), and iron (Fe), organic matter (OM), organic carbon (OC), and calcium (Ca). Soil temperature was measured in situ at the time of collection using a portable thermometer. 

### 7.3. eDNA Extraction from Soil and Nanodrop Spectrophotometry

Eighty-one samples weighing 60 grams each were collected and allotted for eDNA extraction. Each sample was placed in a separate 500 mL sterile beaker, whereupon distilled water was added to create a suspension solution. Fifteen mL from the suspension was immediately subsampled into a 50 mL sterile conical centrifuge tube containing 33 mL of absolute ethanol and 15 mL of 3 M sodium acetate for preservation. The resulting suspension solutions were then stored in a cooler (approximately 0 °C) and were transported to the DNA Barcoding Laboratory at the Institute of Biology Laboratory in UP Diliman for eDNA extraction. The samples were thawed, homogenized, and centrifuged at 8500 rpm for 30 min. The supernatant was discarded and the sediments were homogenized and collected from the centrifuge tube. Sediment of 0.25 g was added to the Lysing Matrix E tube and subsequently subjected to the eDNA extraction protocol of the FastDNA^®^ SPIN Kit for Soil (MP Biomedicals Europe). Extracted eDNA samples were then subjected to spectrophotometry using Nanodrop 2000 (Thermo Scientific) to check for any contamination and DNA purity. The extracted DNA samples were then stored at −20 °C until use. 

### 7.4. Detection via Conventional PCR and qPCR

Each of the 81 extracted eDNA was tested in triplicates using conventional PCR and qPCR to determine the presence of *O. hupensis quadrasi* eDNA. The specific primers and probe to *O. hupensis quadrasi* were designed manually by the alignment of cytochrome *c* oxidase subunit 1 gene sequences of *O. h. quadrasi*, other *Oncomelania* subspecies, and related taxa from other gastropod species from the families Planorbidae, Ampullariidae, Neritidae, Achatinidae, and Thiaridae. The designed primers and probe sequences were then searched against the nucleotide sequence database using BLAST to examine other potential targets. A 187 base pair fragment of *cox1* was targeted using the forward primer OhqCOX1_22-41aF (GCATGTGAGCGGGGCTAGTA) and reverse primer OhqCOX1_189-209aR (AAGCGGAACCAATCAGTTGCC). Positive controls containing pure *cox1* gene of *O. hupensis quadrasi* were used to validate the qPCR setting and primer pair design whereas a negative control without the template was utilized to check for any contamination. For conventional PCR, a 12.50 µL reaction volume per sample was prepared by mixing 7.2 µL of RNase/DNase-free polymerase chain reaction (PCR) grade water (Ambion, Thermo Scientific), 2.5 µL 10X PCR Buffer, 0.64 µL 2.5 mM dNTP mix, 0.25 µL 25 mM MgCl_2_, 0.38 µL µmol each of forward and reverse primers, 0.06 µL 5 U/µL Taq Polymerase (Takara-Clontech), and 1.0 µL of DNA extract with at least 35 ng/µL concentration. PCR was performed in T100™ Thermal Cycler. PCR conditions were the following: 95 °C for initial denaturation for 30 s, 95 °C for denaturation for 5 s, and 60 °C for annealing for 30 s for 50 cycles. PCR products were visualized in a 2% agarose gel (Vivantis Technologies Sdn Bhd) dissolved in 0.5X TBE and stained with 1% ethidium bromide (EtBr) run for 30 mins in 100 v in a horizontal AGE apparatus. *O. hupensis quadrasi* eDNA was also targeted through TaqMan-quantitative real-time PCR using TaqMan System technology in an Applied Biosystems 2720 Thermal Cycler Dice^®^ Real-Time System II (TP 900). The forward and reverse primers used in the conventional PCR were also used. A 10 µL master mix was prepared by mixing 5.5 µL TaqMan Environmental Master Mix (EMM), 1.01 µL of each forward and reverse primers, 0.28 µL TaqMan custom probe (OhqCOX1_67-86P 5’-FAM-GTGCAGAGTTAGGTCAGTCCT-MGB-NFQ-3’), and 2.95 µL of extracted *O. h. quadrasi* eDNA. Samples were then transferred onto well plates and subjected under the following thermal cycling parameters: 95 °C for AmpliTaq Gold^®^, UP enzyme (DNA polymerase) activation under 10 min, 95 °C for denaturation for 15 s, and 60 °C for annealing under 1 min, repeated for 40 cycles (Supplementary Material Figure S1). 

### 7.5. Statistical Analysis

Soil factors and eDNA recovery in all actual and potential snail sites and sampling points were correlated using Pearson’s Correlation available in the SPSS (Statistical Package for the Social Sciences) software v. 16. Pearson’s correlation was utilized to determine the possible linear association between the two variables [soil factor and eDNA detection probability (number of eDNA positive readings in qPCR for a sample)].

## Supplementary Materials

The following are available online at https://www.mdpi.com/2076-0817/8/4/160/s1, Table S1. Malacological survey and conventional Polymerase Chain Reaction (PCR) and TaqMan-quantitative Polymerase Chain Reaction (qPCR) readings of the number of detected environmental DNA (eDNA). (Legend: T- Tapel, S1-Sampling Point Number, PSS1- Potential Snail Site Number, R1-Replicate Number, ASS2- Actual Snail Site Number, M- Magrafil). Table S2. qPCR of *O. hupensis quadrasi* eDNA in barangay Tapel and barangay Magrafil with corresponding Ct (cycle threshold values) and Ct threshold. Table S3. Edaphic factors of all soil samples in Barangay Tapel and Magrafil. Figure S1. TaqMan qPCR amplification plots of *O. hupensis quadrasi*, showing cycle threshold (Ct) levels as indicated by the blue horizontal line; the positive control is indicated by the pink line. The positive result is indicated by amplifications exceeding the threshold line. Each color represents a single sample.

## References

[1] King C.H., Dickman K., Tisch D.J. (2005). Reassessment of the cost of chronic helmintic infection: A meta-Analysis of disability-Related outcomes in endemic schistosomiasis. Lancet.

[2] Steinmann P., Keiser J., Bos R., Tanner M., Utzinger J. (2006). Schistosomiasis and water resources development: Systematic review, meta-Analysis, and estimates of people at risk. Lancet Infect. Dis..

[3] Finkelstein J.L., Schleinitz M.D., Carabin H., McGarvey S.T. (2008). Decision-Model estimation of the age-Specific disability weight for schistosomiasis japonica: A systematic review of the literature. PLoS Negl. Trop. Dis..

[4] Zhou Y., Zheng H., Chen X., Zhang L., Wang K., Guo J., Huang Z., Zhang B., Huang W., Jin K. (2009). The Schistosoma japonicum genome reveals features of host-Parasite interplay. Nature.

[5] Gryseels B., Polman K., Clerinx J., Kestens L. (2006). Human schistosomiasis. Lancet.

[6] Colley D.G., Bustinduy A.L., Secor W.E., King C.H. (2014). Human schistosomiasis. Lancet.

[7] Olveda D.U., Li Y., Olveda R.M., Lam A.K., McManus D.P., Chau T.N.P., Harn D.A., Williams G.M., Gray D.J., Ross A.G.P. (2014). Bilharzia in the Philippines: Past, present, and future. Int. J. Infect. Dis..

[8] Leonardo L., Chigusa Y., Kikuchi M., Kato-Hayashi N., Kawazu S.I., Angeles J.M., Fontanilla I.K., Tabios I.K., Moendeg K., Goto Y. (2016). Schistosomiasis in the Philippines: Challenges and some successes in control. Southeast Asian J. Trop. Med. Public Health.

[9] Blas B. (1991). Handbook for the control of Schistosomiasis japonica. Monogr. Schist Jpn. Infect. Philipp..

[10] Ross A.G.P., Sleigh A.C., Li Y., Davis G.M., Williams G.M., Jiang Z., Feng Z., Manus D.P.M.C. (2001). Schistosomiasis in the People ’ s Republic of China: Prospects and Challenges for the 21st Century. Clin. Microbiol. Rev..

[11] Shi F., Zhang Y., Ye P., Lin J., Cai Y., Shen W., Bickle Q.D., Taylor M.G. (2001). Laboratory and field evaluation of Schistosoma japonicum DNA vaccines in sheep and water buffalo in China. Vaccine.

[12] Blas B.L., Rosales M.I., Lipayon I.L., Yasuraoka K., Matsuda H., Hayashi M. (2004). The schistosomiasis problem in the Philippines: A review. Parasitol. Int..

[13] McGarvey S.T., Carabin H., Balolong E., Bélisle P., Fernandez T., Joseph L., Tallo V., Gonzales R., Tarafder M.R., Alday P. (2006). Cross-Sectional associations between intensity of animal and human infection with Schistosoma japonicum in Western Samar province, Philippines. Bull. World Health Organ..

[14] Conlan J.V., Sripa B., Attwood S., Newton P.N. (2011). A review of parasitic zoonoses in a changing Southeast Asia. Vet. Parasitol..

[15] Wang T.P., Maria V.J., Zhang S.Q., Wang F.F., Wu W.D., Zhang G.H., Pan X.P., Ju Y., Niels Ø. (2005). Transmission of Schistosoma japonicum by humans and domestic animals in the Yangtze River valley, Anhui province, China. Acta Trop..

[16] Ohmae H., Iwanaga Y., Nara T., Matsuda H., Yasuraoka K. (2003). Biological characteristics and control of intermediate snail host of Schistosoma japonicum. Parasitol. Int..

[17] Tanaka H., Tsuji M. (1997). From discovery to eradication of schistosomiasis in Japan: 1847–1996. Int. J. Parasitol..

[18] Minggang C., Zheng F. (1999). Schistosomiasis control in China. Parasitol. Int..

[19] Chua C.J., Tabios I.K., Tamayo P.G., Leonardo L., Fontanilla I.K.C., De Chavez E.R.C., Agatsuma T., Kikuchi M., Kato-Hayashi N., Chigusa Y. (2017). Genetic Comparison of Oncomelania hupensis quadrasi Genetic Comparison of Oncomelania hupensis quad rasi (Möllendorf, 1895) (Gastropoda: Pomatiopsidae), the Intermediate Host of Schistosoma japonicum in the Philippines, Based on 16S Ribosomal RNA Sequence. Sci. Diliman.

[20] Leonardo L., Rivera P., Saniel O., Solon J.A., Chigusa Y., Villacorte E., Chua J.C., Moendeg K., Manalo D., Crisostomo B. (2015). New endemic foci of schistosomiasis infections in the Philippines. Acta Trop..

[21] Pesigan T.P., Hairston N.G., Jauregui J.J., Garcia E.G., Santos A.T., Santos B.C., Besa A.A. (1958). Studies on Schistosoma japonicum infection in the Philippines: 2. The molluscan host. Bull. World Health Organ..

[22] Nihei N., Kanazawa T., Blas B.L., Saitoh Y., Itagaki H., Pangilinan R., Matsuda H., Yasuraoka K. (1998). Soil factors influencing the distribution of Oncomelania quadrasi, the intermediate host of Schistosoma japonicum, on Bohol Island, Philippines. Ann. Trop. Med. Parasitol..

[23] Mcmullen D.B. (1947). The Control Of Schistosomiasis japonica: I. Observations on the habits, ecology and Life Cycle of Oncomelania quadrasi, The Molluscan Intermediate Host Of Schistosoma japonicum in the Philippine Islands. Am. J. Epidemiol..

[24] Pm B., Ingalls J.W. (1948). The molluscan intermediate host and schistosomiasis japonica; observations on the production and rate of emergence of cercariae of Schistosoma japonicum from the molluscan intermediate host, Oncomelania quadrasi. Am. J. Trop. Med. Hyg..

[25] Belizario V.Y., Martinez R.M., de Leon W.U., Esparar D.G., Navarro J.R.P., Villar L.C., Sunico L.S., Velasco L.R., Sison S.M. (2005). Cagayan Valley: A newly described endemic focus for schistosomiasis japonicum in the Philippines. Philipp. J. Intern. Med..

[26] Leonardo L.R., Acosta L.P., Olveda R.M., Aligui G.D.L. (2002). Difficulties and strategies in the control of schistosomiasis in the Philippines. Acta Trop..

[27] Fornillos R.J.C., Fontanilla I.K.C., Chigusa Y., Kikuchi M., Kirinoki M., Kato-Hayashi N., Kawazu S., Angeles J.M., Tabios I.K., Moendeg K. (2019). Infection rate of Schistosoma japonicum in the snail Oncomelania hupensis quadrasi in endemic villages in the Philippines: Need for snail surveillance technique o Title. Trop. Biomed..

[28] Driscoll A.J., Kyle J.L., Remais J. (2005). Development of a novel PCR assay capable of detecting a single Schistosoma japonicum cercaria recovered from Oncomelania hupensis. Parasitology.

[29] Hung Y.W., Remais J. (2008). Quantitative detection of Schistosoma japonicum cercariae in water by real-Time PCR. PLoS Negl. Trop. Dis..

[30] Sato M.O., Rafalimanantsoa A., Ramarokoto C., Rahetilahy A.M., Ravoniarimbinina P., Kawai S., Minamoto T., Sato M., Kirinoki M., Rasolofo V. (2018). Usefulness of environmental DNA for detecting *Schistosoma mansoni* occurrence sites in Madagascar. Int. J. Infect. Dis..

[31] Sengupta M.E., Hellström M., Kariuki H.C., Olsen A., Thomsen P.F., Mejer H., Willerslev E., Mwanje M.T., Madsen H., Kristensen T.K. (2019). Environmental DNA for improved detection and environmental surveillance of schistosomiasis. Proc. Natl. Acad. Sci. USA.

[32] (2019). Google Earth. https://earth.google.com/web/@18.27337328,122.10486135,232.93976988a,88309.32932935d,35y,0h,0t,0r/data=ChMaEQoJL20vMDZqX2hzGAIgASgCNoTitle.

[33] Wilfinger W.W., Mackey K., Chomczynski P. (1997). Effect of pH and ionic strength on the spectrophotometric assessment of nucleic acid purity. Biotechniques.

[34] Desjardins P., Conklin D. (2010). NanoDrop microvolume quantitation of nucleic acids. JoVE.

[35] Schneider J., Valentini A., Dejean T., Montarsi F., Taberlet P., Glaizot O., Fumagalli L. (2016). Detection of invasive mosquito vectors using environmental DNA (eDNA) from water samples. PLoS ONE.

[36] Buxton A.S., Groombridge J.J., Griffiths R.A. (2018). Seasonal variation in environmental DNA detection in sediment and water samples. PLoS ONE.

[37] Ellison S.L.R., English C.A., Burns M.J., Keer J.T. (2006). Routes to improving the reliability of low level DNA analysis using real-time PCR. BMC Biotechnol..

[38] Bohmann K., Evans A., Gilbert M.T.P., Carvalho G.R., Creer S., Knapp M., Douglas W.Y., De Bruyn M. (2014). Environmental DNA for wildlife biology and biodiversity monitoring. Trends Ecol. Evol..

[39] Herder J., Valentini A., Bellemain E., Dejean T., Van Delft J., Thomsen P.F., Taberlet P. (2014). Environmental DNA- A Review of the Possible Applications for the Detection of (Invasive) Species.

[40] Rees H.C., Maddison B.C., Middleditch D.J., Patmore J.R.M., Gough K.C. (2014). The detection of aquatic animal species using environmental DNA—a review of eDNA as a survey tool in ecology. J. Appl. Ecol..

[41] Thomsen P.F., Willerslev E. (2015). Environmental DNA—An emerging tool in conservation for monitoring past and present biodiversity. Biol. Conserv..

[42] Goldberg C.S., Turner C.R., Deiner K., Klymus K.E., Thomsen P.F., Murphy M.A., Spear S.F., McKee A., Oyler-McCance S.J., Cornman R.S. (2016). Critical considerations for the application of environmental DNA methods to detect aquatic species. Methods Ecol. Evol..

[43] Thomsen P.F., Kielgast J., Iversen L.L., Møller P.R., Rasmussen M., Willerslev E. (2012). Detection of a diverse marine fish fauna using environmental DNA from seawater samples. PLoS ONE.

[44] Eichmiller J.J., Best S.E., Sorensen P.W. (2016). Effects of temperature and trophic state on degradation of environmental DNA in lake water. Environ. Sci. Technol..

[45] Johnson P.T.J., Chase J.M., Dosch K.L., Hartson R.B., Gross J.A., Larson D.J., Sutherland D.R., Carpenter S.R. (2007). Aquatic eutrophication promotes pathogenic infection in amphibians. Proc. Natl. Acad. Sci. USA.

[46] Odlare M., Arthurson V., Pell M., Svensson K., Nehrenheim E., Abubaker J. (2011). Land application of organic waste—Effects on the soil ecosystem. Appl. Energy.

[47] Khan S.A., Mulvaney R.L., Ellsworth T.R. (2014). The potassium paradox: Implications for soil fertility, crop production and human health. Renew. Agric. Food Syst..

[48] Shuhaimi-Othman M., Nur-Amalina R., Nadzifah Y. (2012). Toxicity of metals to a freshwater snail, Melanoides tuberculata. Sci. World J..

[49] Gawad S.S.A. (2018). Acute toxicity of some heavy metals to the fresh water snail, Theodoxus niloticus (Reeve, 1856). Egypt. J. Aquat. Res..

[50] Moreno L.I., McCord B.R. (2017). Understanding metal inhibition: The effect of copper (Cu^2+^) on DNA containing samples. Forensic Chem..

[51] Cervantes-Cervantes M.P., Calderón-Salinas J.V., Albores A., Muñoz-Sánchez J.L. (2005). Copper increases the damage to DNA and proteins caused by reactive oxygen species. Biol. Trace Elem. Res..

[52] Jove J.A. (1956). Use of molluscicides in the control of bilharziasis in Venezuela: Equipment and methods of application. Bull. World Health Organ..

[53] Chandiwana S.K., Ndamba J., Makura O., Taylor P. (1987). Field evaluation of controlled release copper glass as a molluscicide in snail control. Trans. R. Soc. Trop. Med. Hyg..

[54] Fahmy S.R., Abdel-Ghaffar F., Bakry F.A., Sayed D.A. (2014). Ecotoxicological effect of sublethal exposure to zinc oxide nanoparticles on freshwater snail Biomphalaria alexandrina. Arch. Environ. Contam. Toxicol..

[55] Ge J.Y., Yang X.H., Yang Y., Feng H.H., Hu X.Y., Wang X.Y. (2014). The Facile Preparation and the Performance of Killing Oncomelania Snails of Zinc Oxide Nanoparticles. Adv. Mater. Res..

[56] Bolyard S. (2012). Fate of Zinc Oxide in Landfill Leachate. Master’s Thesis.

[57] Zhou X.N., Wang L.Y., Chen M.G., Wu X.H., Jiang Q.W., Chen X.Y., Zheng J., Jürg U. (2005). The public health significance and control of schistosomiasis in China—Then and now. Acta Trop..

[58] Mänd R., Tilgar V., Leivits A. (2000). Calcium, snails, and birds: A case study. Web Ecol..

