# Supplementary material for: Analysis of Environmental DNA and Edaphic Factors for the Detection of the Snail Intermediate Host Oncomelania hupensis quadrasi
Source: Pathogens. 2019 Sep 23;8(4):160. doi: 10.3390/pathogens8040160 (PMC6963648; doi:10.3390/pathogens8040160)
Supplement: Supplementary file 1 [file pathogens-08-00160-s001.pdf]

**Supplementary Material**

**Table S1.** Malacological survey and conventional Polymerase Chain Reaction (PCR) and TaqMan-quantitative Polymerase Chain Reaction (qPCR) readings of the number of detected environmental DNA (eDNA). (Legend: T- Tapel, S1-Sampling Point Number, PSS1- Potential Snail Site Number, R1-Replicate Number, ASS2- Actual Snail Site Number, M- Magrafil).

| Sample | Malacological Survey | Conventional PCR (Run 1) | Conventional PCR (Run 2) | Conventional PCR (Run 3) | qPCR (Run 1) | qPCR (Run 2) | qPCR (Run 3) | Total number of detected using qPCR |
|---|---|---|---|---|---|---|---|---|
| TS1-PSS1 (R1) | - | - | - | - | + | - | + | 3/9 |
| TS1-PSS1 (R2) | - | - | - | - | - | - | + | |
| TS1-PSS1 (R3) | - | - | - | - | - | - | - | |
| TS1-ASS2 (R1) | + | - | - | - | - | - | + | 4/9 |
| TS1-ASS2 (R2) | + | - | - | - | - | - | + | |
| TS1-ASS2 (R3) | + | - | - | - | + | - | + | |
| TS1-PSS3 (R1) | - | - | - | - | - | - | + | 4/9 |
| TS1-PSS3 (R2) | - | - | - | - | + | - | + | |
| TS1-PSS3 (R3) | - | - | - | - | + | - | - | |
| TS2-PSS1 (R1) | - | - | - | - | - | + | - | 2/9 |
| TS2-PSS1 (R2) | - | - | - | - | - | - | - | |
| TS2-PSS1 (R3) | - | - | - | - | + | - | - | |
| TS2-ASS2 (R1) | + | - | - | - | + | + | - | 2/9 |
| TS2-ASS2 (R2) | + | - | - | - | - | - | - | |
| TS2-ASS2 (R3) | + | - | - | - | - | - | - | |
| TS2-PSS3 (R1) | - | - | - | - | + | + | + | 9/9 |
| TS2-PSS3 (R2) | - | - | - | - | + | + | + | |
| TS2-PSS3 (R3) | - | - | - | - | + | + | + | |
| TS3-PSS1 (R1) | - | - | - | - | - | - | - | 0/9 |
| TS3-PSS1 (R2) | - | - | - | - | - | - | - | |
| TS3-PSS1 (R3) | - | - | - | - | - | - | - | |
| TS3-ASS2 (R1) | + | - | - | - | + | + | - | 6/9 |
| TS3-ASS2 (R2) | + | - | - | - | + | + | + | |
| TS3-ASS2 (R3) | + | - | - | - | - | + | - | |
| TS3-PSS3 (R1) | - | - | - | - | - | - | - | 1/9 |
| TS3-PSS3 (R2) | - | - | - | - | + | - | - | |
| TS3-PSS3 (R3) | - | - | - | - | - | - | - | |
| TS4-PSS1 (R1) | - | - | - | - | + | - | - | 3/9 |
| TS4-PSS1 (R2) | - | - | - | - | + | - | - | |
| TS4-PSS1 (R3) | - | - | - | - | - | + | - | |
| TS4-ASS2 (R1) | + | - | - | - | - | - | - | 0/9 |
| TS4-ASS2 (R2) | + | - | - | - | - | - | - | |
| TS4-ASS2 (R3) | + | - | - | - | - | - | - | |
| TS4-PSS3 (R1) | - | - | - | - | + | - | - | 1/9 |
| TS4-PSS3 (R2) | - | - | - | - | - | - | - | |
| TS4-PSS3 (R3) | - | - | - | - | - | - | - | |
| TS5-PSS1 (R1) | - | - | - | - | - | - | - | 0/9 |
| TS5-PSS1 (R2) | - | - | - | - | - | - | - | |

| | | | | | | | | |
|---|---|---|---|---|---|---|---|---|
| TS5-PSS1 (R3) | - | - | - | - | - | - | - | |
| TS5-ASS2 (R1) | + | - | - | - | - | - | - | 0/9 |
| TS5-ASS2 (R2) | + | - | - | - | - | - | - | |
| TS5-ASS2 (R3) | + | - | - | - | - | - | - | |
| TS5-PSS3 (R1) | - | - | - | - | - | - | - | 0/9 |
| TS5-PSS3 (R2) | - | - | - | - | - | - | - | |
| TS5-PSS3 (R3) | - | - | - | - | - | - | - | |
| MS6-PSS1 (R1) | - | - | - | - | - | - | - | 1/9 |
| MS6-PSS1 (R2) | - | - | - | - | - | - | - | |
| MS6-PSS1 (R3) | - | - | - | - | + | - | - | |
| MS6-ASS2 (R1) | + | - | - | - | - | - | - | 3/9 |
| MS6-ASS2 (R2) | + | - | - | - | - | - | - | |
| MS6-ASS2 (R3) | + | - | - | - | + | + | + | |
| MS6-PSS3 (R1) | - | - | - | - | + | + | + | 9/9 |
| MS6-PSS3 (R2) | - | - | - | - | + | + | + | |
| MS6-PSS3 (R3) | - | - | - | - | + | + | + | |
| MS7-PSS1 (R1) | - | - | - | - | + | + | + | 9/9 |
| MS7-PSS1 (R2) | - | - | - | - | + | + | + | |
| MS7-PSS1 (R3) | - | - | - | - | + | + | + | |
| MS7-ASS2 (R1) | + | - | - | - | - | + | + | 6/9 |
| MS7-ASS2 (R2) | + | - | - | - | - | + | + | |
| MS7-ASS2 (R3) | + | - | - | - | - | + | + | |
| MS7-PSS3 (R1) | - | - | - | - | + | + | + | 9/9 |
| MS7-PSS3 (R2) | - | - | - | - | + | + | + | |
| MS7-PSS3 (R3) | - | - | - | - | + | + | + | |
| MS8-PSS1 (R1) | - | - | - | - | - | + | + | 6/9 |
| MS8-PSS1 (R2) | - | - | - | - | - | + | + | |
| MS8-PSS1 (R3) | - | - | - | - | - | + | + | |
| MS8-ASS2 (R1) | + | - | - | - | - | + | + | 6/9 |
| MS8-ASS2 (R2) | + | - | - | - | - | + | + | |
| MS8-ASS3 (R3) | + | - | - | - | - | + | + | |
| MS8-PSS3 (R1) | - | - | - | - | - | + | + | 6/9 |
| MS8-PSS3 (R2) | - | - | - | - | - | + | + | |
| MS8-PSS3 (R3) | - | - | - | - | - | + | + | |
| MS9-PSS1 (R1) | - | - | - | - | - | + | + | 6/9 |
| MS9-PSS1 (R2) | - | - | - | - | - | + | + | |
| MS9-PSS1 (R3) | - | - | - | - | - | + | + | |
| MS9-ASS2 (R1) | + | - | - | - | - | + | + | 7/9 |
| MS9-ASS2 (R2) | + | - | - | - | - | + | + | |
| MS9-ASS3 (R3) | + | - | - | - | + | + | + | |
| MS9-PSS3 (R1) | - | - | - | - | + | + | + | 8/9 |
| MS9-PSS3 (R2) | - | - | - | - | + | + | + | |
| MS9-PSS3 (R3) | - | - | - | - | + | + | - | |

**Table S2.** qPCR of *O. hupensis quadrasi* eDNA in barangay Tapel and barangay Magrafil with corresponding Ct (cycle threshold values) and Ct threshold.

| Sample Name | Run | Target Name | Task | Reporter | Quencher | Ct | Ct Threshold |
|---|---|---|---|---|---|---|---|
| Negative | 1 | cox1 | NTC | FAM | NFQ-MGB | Undetermined | 0.006316 |
| | 2 | cox1 | NTC | FAM | NFQ-MGB | Undetermined | 0.018889 |
| | 3 | cox1 | NTC | FAM | NFQ-MGB | Undetermined | 0.008638 |
| TS1-PSS1 (R1) | 1 | cox1 | UNKNOWN | FAM | NFQ-MGB | 31.82 | 0.006316 |
| | 2 | cox1 | UNKNOWN | FAM | NFQ-MGB | Undetermined | 0.008638 |
| | 3 | cox1 | UNKNOWN | FAM | NFQ-MGB | 31.93 | 0.008638 |
| TS1-PSS1 (R2) | 1 | cox1 | UNKNOWN | FAM | NFQ-MGB | Undetermined | 0.006316 |
| | 2 | cox1 | UNKNOWN | FAM | NFQ-MGB | Undetermined | 0.008638 |
| | 3 | cox1 | UNKNOWN | FAM | NFQ-MGB | 31.08 | 0.008638 |
| TS1-PSS1 (R3) | 1 | cox1 | UNKNOWN | FAM | NFQ-MGB | Undetermined | 0.006316 |
| | 2 | cox1 | UNKNOWN | FAM | NFQ-MGB | Undetermined | 0.008638 |
| | 3 | cox1 | UNKNOWN | FAM | NFQ-MGB | Undetermined | 0.008638 |
| TS1-ASS2 (R1) | 1 | cox1 | UNKNOWN | FAM | NFQ-MGB | Undetermined | 0.006316 |
| | 2 | cox1 | UNKNOWN | FAM | NFQ-MGB | Undetermined | 0.008638 |
| | 3 | cox1 | UNKNOWN | FAM | NFQ-MGB | 31.30 | 0.008638 |
| TS1-ASS2 (R2) | 1 | cox1 | UNKNOWN | FAM | NFQ-MGB | Undetermined | 0.006316 |
| | 2 | cox1 | UNKNOWN | FAM | NFQ-MGB | Undetermined | 0.008638 |
| | 3 | cox1 | UNKNOWN | FAM | NFQ-MGB | 32.02 | 0.008638 |
| TS1-ASS2 (R3) | 1 | cox1 | UNKNOWN | FAM | NFQ-MGB | 31.93 | 0.006316 |

| | | | | | | | |
|---|---|---|---|---|---|---|---|
| | 2 | cox1 | UNKNOWN | FAM | NFQ-MGB | Undetermined | 0.008638 |
| | 3 | cox1 | UNKNOWN | FAM | NFQ-MGB | 30.20 | 0.008638 |
| TS1-PSS3 (R1) | 1 | cox1 | UNKNOWN | FAM | NFQ-MGB | Undetermined | 0.006316 |
| | 2 | cox1 | UNKNOWN | FAM | NFQ-MGB | Undetermined | 0.008638 |
| | 3 | cox1 | UNKNOWN | FAM | NFQ-MGB | 32.14 | 0.008638 |
| TS1-PSS3 (R2) | 1 | cox1 | UNKNOWN | FAM | NFQ-MGB | 32.75 | 0.006316 |
| | 2 | cox1 | UNKNOWN | FAM | NFQ-MGB | Undetermined | 0.008638 |
| | 3 | cox1 | UNKNOWN | FAM | NFQ-MGB | 30.50 | 0.008638 |
| TS1-PSS3 (R3) | 1 | cox1 | UNKNOWN | FAM | NFQ-MGB | 29.52 | 0.006316 |
| | 2 | cox1 | UNKNOWN | FAM | NFQ-MGB | Undetermined | 0.008638 |
| | 3 | cox1 | UNKNOWN | FAM | NFQ-MGB | Undetermined | 0.008638 |
| TS2-PSS1 (R1) | 1 | cox1 | UNKNOWN | FAM | NFQ-MGB | Undetermined | 0.006316 |
| | 2 | cox1 | UNKNOWN | FAM | NFQ-MGB | 32.76 | 0.008638 |
| | 3 | cox1 | UNKNOWN | FAM | NFQ-MGB | Undetermined | 0.008638 |
| TS2-PSS1 (R2) | 1 | cox1 | UNKNOWN | FAM | NFQ-MGB | Undetermined | 0.006316 |
| | 2 | cox1 | UNKNOWN | FAM | NFQ-MGB | Undetermined | 0.008638 |
| | 3 | cox1 | UNKNOWN | FAM | NFQ-MGB | Undetermined | 0.008638 |
| TS2-PSS1 (R3) | 1 | cox1 | UNKNOWN | FAM | NFQ-MGB | 30.01 | 0.006316 |
| | 2 | cox1 | UNKNOWN | FAM | NFQ-MGB | Undetermined | 0.008638 |
| | 3 | cox1 | UNKNOWN | FAM | NFQ-MGB | Undetermined | 0.008638 |
| TS2-ASS2 (R1) | 1 | cox1 | UNKNOWN | FAM | NFQ-MGB | 37.19 | 0.006316 |

| | | | | | | | |
|---|---|---|---|---|---|---|---|
| | 2 | cox1 | UNKNOWN | FAM | NFQ-MGB | 29.66 | 0.008638 |
| | 3 | cox1 | UNKNOWN | FAM | NFQ-MGB | Undetermined | 0.008638 |
| TS2-ASS2 (R2) | 1 | cox1 | UNKNOWN | FAM | NFQ-MGB | Undetermined | 0.006316 |
| | 2 | cox1 | UNKNOWN | FAM | NFQ-MGB | Undetermined | 0.008638 |
| | 3 | cox1 | UNKNOWN | FAM | NFQ-MGB | Undetermined | 0.008638 |
| TS2-ASS2 (R3) | 1 | cox1 | UNKNOWN | FAM | NFQ-MGB | Undetermined | 0.006316 |
| | 2 | cox1 | UNKNOWN | FAM | NFQ-MGB | Undetermined | 0.008638 |
| | 3 | cox1 | UNKNOWN | FAM | NFQ-MGB | Undetermined | 0.008638 |
| TS2-PSS3 (R1) | 1 | cox1 | UNKNOWN | FAM | NFQ-MGB | 30.14 | 0.006316 |
| | 2 | cox1 | UNKNOWN | FAM | NFQ-MGB | 27.77 | 0.008638 |
| | 3 | cox1 | UNKNOWN | FAM | NFQ-MGB | 29.0 | 0.008638 |
| TS2-PSS3 (R2) | 1 | cox1 | UNKNOWN | FAM | NFQ-MGB | 28.18 | 0.006316 |
| | 2 | cox1 | UNKNOWN | FAM | NFQ-MGB | 27.72 | 0.008638 |
| | 3 | cox1 | UNKNOWN | FAM | NFQ-MGB | 28.03 | 0.008638 |
| TS2-PSS3 (R3) | 1 | cox1 | UNKNOWN | FAM | NFQ-MGB | 29.01 | 0.006316 |
| | 2 | cox1 | UNKNOWN | FAM | NFQ-MGB | 28.03 | 0.008638 |
| | 3 | cox1 | UNKNOWN | FAM | NFQ-MGB | 29.16 | 0.008638 |
| TS3-PSS1 (R1) | 1 | cox1 | UNKNOWN | FAM | NFQ-MGB | Undetermined | 0.006316 |
| | 2 | cox1 | UNKNOWN | FAM | NFQ-MGB | Undetermined | 0.008638 |
| | 3 | cox1 | UNKNOWN | FAM | NFQ-MGB | Undetermined | 0.008638 |
| TS3-PSS1 (R2) | 1 | cox1 | UNKNOWN | FAM | NFQ-MGB | Undetermined | 0.006316 |

| | | | | | | | |
|---|---|---|---|---|---|---|---|
| | 2 | cox1 | UNKNOWN | FAM | NFQ-MGB | Undetermined | 0.008638 |
| | 3 | cox1 | UNKNOWN | FAM | NFQ-MGB | Undetermined | 0.008638 |
| TS3-PSS1 (R3) | 1 | cox1 | UNKNOWN | FAM | NFQ-MGB | Undetermined | 0.006316 |
| | 2 | cox1 | UNKNOWN | FAM | NFQ-MGB | Undetermined | 0.008638 |
| | 3 | cox1 | UNKNOWN | FAM | NFQ-MGB | Undetermined | 0.008638 |
| TS3-ASS2 (R1) | 1 | cox1 | UNKNOWN | FAM | NFQ-MGB | 30.51 | 0.006316 |
| | 2 | cox1 | UNKNOWN | FAM | NFQ-MGB | 31.48 | 0.008638 |
| | 3 | cox1 | UNKNOWN | FAM | NFQ-MGB | Undetermined | 0.008638 |
| TS3-ASS2 (R2) | 1 | cox1 | UNKNOWN | FAM | NFQ-MGB | 29.29 | 0.006316 |
| | 2 | cox1 | UNKNOWN | FAM | NFQ-MGB | 27.79 | 0.008638 |
| | 3 | cox1 | UNKNOWN | FAM | NFQ-MGB | 30.87 | 0.008638 |
| TS3-ASS2 (R3) | 1 | cox1 | UNKNOWN | FAM | NFQ-MGB | Undetermined | 0.006316 |
| | 2 | cox1 | UNKNOWN | FAM | NFQ-MGB | 17.38 | 0.008638 |
| | 3 | cox1 | UNKNOWN | FAM | NFQ-MGB | Undetermined | 0.008638 |
| TS3-PSS3 (R1) | 1 | cox1 | UNKNOWN | FAM | NFQ-MGB | Undetermined | 0.006316 |
| | 2 | cox1 | UNKNOWN | FAM | NFQ-MGB | Undetermined | 0.008638 |
| | 3 | cox1 | UNKNOWN | FAM | NFQ-MGB | Undetermined | 0.008638 |
| TS3-PSS3 (R2) | 1 | cox1 | UNKNOWN | FAM | NFQ-MGB | 15.86 | 0.006316 |
| | 2 | cox1 | UNKNOWN | FAM | NFQ-MGB | Undetermined | 0.008638 |
| | 3 | cox1 | UNKNOWN | FAM | NFQ-MGB | Undetermined | 0.008638 |
| TS3-PSS3 (R3) | 1 | cox1 | UNKNOWN | FAM | NFQ-MGB | Undetermined | 0.006316 |

| | | | | | | | |
|---|---|---|---|---|---|---|---|
| | 2 | cox1 | UNKNOWN | FAM | NFQ-MGB | Undetermined | 0.008638 |
| | 3 | cox1 | UNKNOWN | FAM | NFQ-MGB | Undetermined | 0.008638 |
| TS4-PSS1 (R1) | 1 | cox1 | UNKNOWN | FAM | NFQ-MGB | 5.81 | 0.006316 |
| | 2 | cox1 | UNKNOWN | FAM | NFQ-MGB | Undetermined | 0.008638 |
| | 3 | cox1 | UNKNOWN | FAM | NFQ-MGB | Undetermined | 0.008638 |
| TS4-PSS1 (R2) | 1 | cox1 | UNKNOWN | FAM | NFQ-MGB | 26.72 | 0.006316 |
| | 2 | cox1 | UNKNOWN | FAM | NFQ-MGB | Undetermined | 0.008638 |
| | 3 | cox1 | UNKNOWN | FAM | NFQ-MGB | Undetermined | 0.008638 |
| TS4-PSS1 (R3) | 1 | cox1 | UNKNOWN | FAM | NFQ-MGB | Undetermined | 0.006316 |
| | 2 | cox1 | UNKNOWN | FAM | NFQ-MGB | 37.97 | 0.008638 |
| | 3 | cox1 | UNKNOWN | FAM | NFQ-MGB | Undetermined | 0.008638 |
| TS4-ASS2 (R1) | 1 | cox1 | UNKNOWN | FAM | NFQ-MGB | Undetermined | 0.008638 |
| | 2 | cox1 | UNKNOWN | FAM | NFQ-MGB | Undetermined | 0.008638 |
| | 3 | cox1 | UNKNOWN | FAM | NFQ-MGB | Undetermined | 0.008638 |
| Positive | 1 | cox1 | UNKNOWN | FAM | NFQ-MGB | 13.48 | 0.006316 |
| | 2 | cox1 | UNKNOWN | FAM | NFQ-MGB | 14.04 | 0.008638 |
| | 3 | cox1 | UNKNOWN | FAM | NFQ-MGB | 14.69 | 0.008638 |
| Negative | 1 | cox1 | NTC | FAM | NFQ-MGB | Undetermined | 0.022037 |
| | 2 | cox1 | NTC | FAM | NFQ-MGB | Undetermined | 0.022037 |
| | 3 | cox1 | NTC | FAM | NFQ-MGB | Undetermined | 0.022037 |
| TS4-ASS2 (R1) | 1 | cox1 | UNKNOWN | FAM | NFQ-MGB | Undetermined | 0.022037 |

| | | | | | | | |
|---|---|---|---|---|---|---|---|
| | 2 | cox1 | UNKNOWN | FAM | NFQ-MGB | Undetermined | 0.022037 |
| | 3 | cox1 | UNKNOWN | FAM | NFQ-MGB | Undetermined | 0.022037 |
| TS4-ASS2 (R2) | 1 | cox1 | UNKNOWN | FAM | NFQ-MGB | Undetermined | 0.022037 |
| | 2 | cox1 | UNKNOWN | FAM | NFQ-MGB | Undetermined | 0.022037 |
| | 3 | cox1 | UNKNOWN | FAM | NFQ-MGB | Undetermined | 0.022037 |
| TS4-ASS2 (R3) | 1 | cox1 | UNKNOWN | FAM | NFQ-MGB | Undetermined | 0.022037 |
| | 2 | cox1 | UNKNOWN | FAM | NFQ-MGB | Undetermined | 0.022037 |
| | 3 | cox1 | UNKNOWN | FAM | NFQ-MGB | Undetermined | 0.022037 |
| TS4-PSS3 (R1) | 1 | cox1 | UNKNOWN | FAM | NFQ-MGB | 34.56 | 0.022037 |
| | 2 | cox1 | UNKNOWN | FAM | NFQ-MGB | Undetermined | 0.022037 |
| | 3 | cox1 | UNKNOWN | FAM | NFQ-MGB | Undetermined | 0.022037 |
| TS4-PSS3 (R2) | 1 | cox1 | UNKNOWN | FAM | NFQ-MGB | Undetermined | 0.022037 |
| | 2 | cox1 | UNKNOWN | FAM | NFQ-MGB | Undetermined | 0.022037 |
| | 3 | cox1 | UNKNOWN | FAM | NFQ-MGB | Undetermined | 0.022037 |
| TS4-PSS3 (R3) | 1 | cox1 | UNKNOWN | FAM | NFQ-MGB | Undetermined | 0.022037 |
| | 2 | cox1 | UNKNOWN | FAM | NFQ-MGB | Undetermined | 0.022037 |
| | 3 | cox1 | UNKNOWN | FAM | NFQ-MGB | Undetermined | 0.022037 |
| TS5-PSS1(R1) | 1 | cox1 | UNKNOWN | FAM | NFQ-MGB | Undetermined | 0.022037 |
| | 2 | cox1 | UNKNOWN | FAM | NFQ-MGB | Undetermined | 0.022037 |
| | 3 | cox1 | UNKNOWN | FAM | NFQ-MGB | Undetermined | 0.022037 |
| TS5-PSS1(R2) | 1 | cox1 | UNKNOWN | FAM | NFQ-MGB | Undetermined | 0.022037 |

| | | | | | | | |
|---|---|---|---|---|---|---|---|
| | 2 | cox1 | UNKNOWN | FAM | NFQ-MGB | Undetermined | 0.022037 |
| | 3 | cox1 | UNKNOWN | FAM | NFQ-MGB | Undetermined | 0.022037 |
| TS5-PSS1(R3) | 1 | cox1 | UNKNOWN | FAM | NFQ-MGB | Undetermined | 0.022037 |
| | 2 | cox1 | UNKNOWN | FAM | NFQ-MGB | Undetermined | 0.022037 |
| | 3 | cox1 | UNKNOWN | FAM | NFQ-MGB | Undetermined | 0.022037 |
| TS5-ASS2 (R1) | 1 | cox1 | UNKNOWN | FAM | NFQ-MGB | Undetermined | 0.022037 |
| | 2 | cox1 | UNKNOWN | FAM | NFQ-MGB | Undetermined | 0.022037 |
| | 3 | cox1 | UNKNOWN | FAM | NFQ-MGB | Undetermined | 0.022037 |
| TS5-ASS2 (R2) | 1 | cox1 | UNKNOWN | FAM | NFQ-MGB | Undetermined | 0.022037 |
| | 2 | cox1 | UNKNOWN | FAM | NFQ-MGB | Undetermined | 0.022037 |
| | 3 | cox1 | UNKNOWN | FAM | NFQ-MGB | Undetermined | 0.022037 |
| TS5-ASS2 (R3) | 1 | cox1 | UNKNOWN | FAM | NFQ-MGB | Undetermined | 0.022037 |
| | 2 | cox1 | UNKNOWN | FAM | NFQ-MGB | Undetermined | 0.022037 |
| | 3 | cox1 | UNKNOWN | FAM | NFQ-MGB | Undetermined | 0.022037 |
| TS5-PSS3 (R1) | 1 | cox1 | UNKNOWN | FAM | NFQ-MGB | Undetermined | 0.022037 |
| | 2 | cox1 | UNKNOWN | FAM | NFQ-MGB | Undetermined | 0.022037 |
| | 3 | cox1 | UNKNOWN | FAM | NFQ-MGB | Undetermined | 0.022037 |
| TS5- PSS3 (R2) | 1 | cox1 | UNKNOWN | FAM | NFQ-MGB | Undetermined | 0.022037 |
| | 2 | cox1 | UNKNOWN | FAM | NFQ-MGB | Undetermined | 0.022037 |
| | 3 | cox1 | UNKNOWN | FAM | NFQ-MGB | Undetermined | 0.022037 |
| TS5- PSS3 (R3) | 1 | cox1 | UNKNOWN | FAM | NFQ-MGB | Undetermined | 0.022037 |

| | | | | | | | |
|---|---|---|---|---|---|---|---|
| | 2 | cox1 | UNKNOWN | FAM | NFQ-MGB | Undetermined | 0.022037 |
| | 3 | cox1 | UNKNOWN | FAM | NFQ-MGB | Undetermined | 0.022037 |
| MS6-PSS1 (R1) | 1 | cox1 | UNKNOWN | FAM | NFQ-MGB | Undetermined | 0.022037 |
| | 2 | cox1 | UNKNOWN | FAM | NFQ-MGB | Undetermined | 0.022037 |
| | 3 | cox1 | UNKNOWN | FAM | NFQ-MGB | Undetermined | 0.022037 |
| MS6-PSS1 (R2) | 1 | cox1 | UNKNOWN | FAM | NFQ-MGB | Undetermined | 0.022037 |
| | 2 | cox1 | UNKNOWN | FAM | NFQ-MGB | Undetermined | 0.022037 |
| | 3 | cox1 | UNKNOWN | FAM | NFQ-MGB | Undetermined | 0.022037 |
| MS6-PSS1 (R3) | 1 | cox1 | UNKNOWN | FAM | NFQ-MGB | 38.10 | 0.022037 |
| | 2 | cox1 | UNKNOWN | FAM | NFQ-MGB | Undetermined | 0.022037 |
| | 3 | cox1 | UNKNOWN | FAM | NFQ-MGB | Undetermined | 0.022037 |
| MS6-ASS2 (R1) | 1 | cox1 | UNKNOWN | FAM | NFQ-MGB | Undetermined | 0.022037 |
| | 2 | cox1 | UNKNOWN | FAM | NFQ-MGB | Undetermined | 0.022037 |
| | 3 | cox1 | UNKNOWN | FAM | NFQ-MGB | Undetermined | 0.022037 |
| MS6-ASS2 (R2) | 1 | cox1 | UNKNOWN | FAM | NFQ-MGB | Undetermined | 0.022037 |
| | 2 | cox1 | UNKNOWN | FAM | NFQ-MGB | Undetermined | 0.022037 |
| | 3 | cox1 | UNKNOWN | FAM | NFQ-MGB | Undetermined | 0.022037 |
| MS6-ASS2 (R3) | 1 | cox1 | UNKNOWN | FAM | NFQ-MGB | 34.47 | 0.022037 |
| | 2 | cox1 | UNKNOWN | FAM | NFQ-MGB | 31.86 | 0.022037 |
| | 3 | cox1 | UNKNOWN | FAM | NFQ-MGB | 32.04 | 0.022037 |
| MS6-PSS3 (R1) | 1 | cox1 | UNKNOWN | FAM | NFQ-MGB | 33.73 | 0.022037 |

| | | | | | | | |
|---|---|---|---|---|---|---|---|
| | 2 | cox1 | UNKNOWN | FAM | NFQ-MGB | 36.73 | 0.022037 |
| | 3 | cox1 | UNKNOWN | FAM | NFQ-MGB | 33.06 | 0.022037 |
| MS6-PSS3 (R2) | 1 | cox1 | UNKNOWN | FAM | NFQ-MGB | 32.21 | 0.022037 |
| | 2 | cox1 | UNKNOWN | FAM | NFQ-MGB | 33.01 | 0.022037 |
| | 3 | cox1 | UNKNOWN | FAM | NFQ-MGB | 32.94 | 0.022037 |
| MS6-PSS3 (R3) | 1 | cox1 | UNKNOWN | FAM | NFQ-MGB | 34.48 | 0.022037 |
| | 2 | cox1 | UNKNOWN | FAM | NFQ-MGB | 32.46 | 0.022037 |
| | 3 | cox1 | UNKNOWN | FAM | NFQ-MGB | 34.60 | 0.022037 |
| MS7-PSS1 (R1) | 1 | cox1 | UNKNOWN | FAM | NFQ-MGB | 36.02 | 0.022037 |
| | 2 | cox1 | UNKNOWN | FAM | NFQ-MGB | 34.38 | 0.022037 |
| | 3 | cox1 | UNKNOWN | FAM | NFQ-MGB | 37.02 | 0.022037 |
| MS7-PSS1 (R2) | 1 | cox1 | UNKNOWN | FAM | NFQ-MGB | 32.40 | 0.022037 |
| | 2 | cox1 | UNKNOWN | FAM | NFQ-MGB | 32.19 | 0.022037 |
| | 3 | cox1 | UNKNOWN | FAM | NFQ-MGB | 32.68 | 0.022037 |
| MS7-PSS1 (R3) | 1 | cox1 | UNKNOWN | FAM | NFQ-MGB | 34.11 | 0.022037 |
| | 2 | cox1 | UNKNOWN | FAM | NFQ-MGB | 34.24 | 0.022037 |
| | 3 | cox1 | UNKNOWN | FAM | NFQ-MGB | 35.77 | 0.022037 |
| MS7-ASS2 (R1) | 1 | cox1 | UNKNOWN | FAM | NFQ-MGB | Undetermined | 0.018889 |
| | 2 | cox1 | UNKNOWN | FAM | NFQ-MGB | 29.32 | 0.018889 |
| | 3 | cox1 | UNKNOWN | FAM | NFQ-MGB | 29.11 | 0.018889 |
| MS7-ASS2 (R2) | 1 | cox1 | UNKNOWN | FAM | NFQ-MGB | Undetermined | 0.018889 |

| | | | | | | | |
|---|---|---|---|---|---|---|---|
| | 2 | cox1 | UNKNOWN | FAM | NFQ-MGB | 29.57 | 0.018889 |
| | 3 | cox1 | UNKNOWN | FAM | NFQ-MGB | 29.83 | 0.018889 |
| MS7-ASS2 (R3) | 1 | cox1 | UNKNOWN | FAM | NFQ-MGB | Undetermined | 0.018889 |
| | 2 | cox1 | UNKNOWN | FAM | NFQ-MGB | 24.90 | 0.018889 |
| | 3 | cox1 | UNKNOWN | FAM | NFQ-MGB | 24.69 | 0.018889 |
| MS7-PSS3 (R1) | 1 | cox1 | UNKNOWN | FAM | NFQ-MGB | 29.98 | 0.022037 |
| | 2 | cox1 | UNKNOWN | FAM | NFQ-MGB | 29.67 | 0.022037 |
| | 3 | cox1 | UNKNOWN | FAM | NFQ-MGB | 30.26 | 0.022037 |
| MS7-PSS3 (R2) | 1 | cox1 | UNKNOWN | FAM | NFQ-MGB | 32.92 | 0.022037 |
| | 2 | cox1 | UNKNOWN | FAM | NFQ-MGB | 32.75 | 0.022037 |
| | 3 | cox1 | UNKNOWN | FAM | NFQ-MGB | 34.06 | 0.022037 |
| MS7-PSS3 (R3) | 1 | cox1 | UNKNOWN | FAM | NFQ-MGB | 32.95 | 0.022037 |
| | 2 | cox1 | UNKNOWN | FAM | NFQ-MGB | 31.88 | 0.022037 |
| | 3 | cox1 | UNKNOWN | FAM | NFQ-MGB | 33.70 | 0.022037 |
| Positive | 1 | cox1 | UNKNOWN | FAM | NFQ-MGB | 15.88 | 0.022037 |
| | 2 | cox1 | UNKNOWN | FAM | NFQ-MGB | 15.25 | 0.022037 |
| | 3 | cox1 | UNKNOWN | FAM | NFQ-MGB | 20.02 | 0.022037 |
| Negative Control | 1 | cox1 | NTC | FAM | NFQ-MGB | Undetermined | 0.018889 |
| | 2 | cox1 | NTC | FAM | NFQ-MGB | Undetermined | 0.018889 |
| | 3 | cox1 | NTC | FAM | NFQ-MGB | Undetermined | 0.018889 |
| MS8-PSS1 (R1) | 1 | cox1 | UNKNOWN | FAM | NFQ-MGB | Undetermined | 0.018889 |

| | | | | | | | |
|---|---|---|---|---|---|---|---|
| | 2 | cox1 | UNKNOWN | FAM | NFQ-MGB | 28.87 | 0.018889 |
| | 3 | cox1 | UNKNOWN | FAM | NFQ-MGB | 29.03 | 0.018889 |
| MS8-PSS1 (R2) | 1 | cox1 | UNKNOWN | FAM | NFQ-MGB | Undetermined | 0.018889 |
| | 2 | cox1 | UNKNOWN | FAM | NFQ-MGB | 30.00 | 0.018889 |
| | 3 | cox1 | UNKNOWN | FAM | NFQ-MGB | 29.91 | 0.018889 |
| MS8-PSS1 (R3) | 1 | cox1 | UNKNOWN | FAM | NFQ-MGB | Undetermined | 0.018889 |
| | 2 | cox1 | UNKNOWN | FAM | NFQ-MGB | 29.93 | 0.018889 |
| | 3 | cox1 | UNKNOWN | FAM | NFQ-MGB | 29.32 | 0.018889 |
| MS8-ASS2 (R1) | 1 | cox1 | UNKNOWN | FAM | NFQ-MGB | Undetermined | 0.018889 |
| | 2 | cox1 | UNKNOWN | FAM | NFQ-MGB | 29.95 | 0.018889 |
| | 3 | cox1 | UNKNOWN | FAM | NFQ-MGB | 29.99 | 0.018889 |
| MS8-ASS2 (R2) | 1 | cox1 | UNKNOWN | FAM | NFQ-MGB | Undetermined | 0.018889 |
| | 2 | cox1 | UNKNOWN | FAM | NFQ-MGB | 29.71 | 0.018889 |
| | 3 | cox1 | UNKNOWN | FAM | NFQ-MGB | 30.76 | 0.018889 |
| MS8-ASS2 (R3) | 1 | cox1 | UNKNOWN | FAM | NFQ-MGB | Undetermined | 0.018889 |
| | 2 | cox1 | UNKNOWN | FAM | NFQ-MGB | 29.74 | 0.018889 |
| | 3 | cox1 | UNKNOWN | FAM | NFQ-MGB | 30.45 | 0.018889 |
| MS8-PSS3 (R1) | 1 | cox1 | UNKNOWN | FAM | NFQ-MGB | Undetermined | 0.018889 |
| | 2 | cox1 | UNKNOWN | FAM | NFQ-MGB | 30.93 | 0.018889 |
| | 3 | cox1 | UNKNOWN | FAM | NFQ-MGB | 30.92 | 0.018889 |
| MS8-PSS3 (R2) | 1 | cox1 | UNKNOWN | FAM | NFQ-MGB | Undetermined | 0.018889 |

| | | | | | | | |
|---|---|---|---|---|---|---|---|
| | 2 | cox1 | UNKNOWN | FAM | NFQ-MGB | 30.44 | 0.018889 |
| | 3 | cox1 | UNKNOWN | FAM | NFQ-MGB | 30.82 | 0.018889 |
| MS8-PSS3 (R3) | 1 | cox1 | UNKNOWN | FAM | NFQ-MGB | Undetermined | 0.018889 |
| | 2 | cox1 | UNKNOWN | FAM | NFQ-MGB | 30.56 | 0.018889 |
| | 1 | cox1 | UNKNOWN | FAM | NFQ-MGB | 30.71 | 0.018889 |
| MS9-PSS1 (R1) | 1 | cox1 | UNKNOWN | FAM | NFQ-MGB | Undetermined | 0.018889 |
| | 2 | cox1 | UNKNOWN | FAM | NFQ-MGB | 27.75 | 0.018889 |
| | 3 | cox1 | UNKNOWN | FAM | NFQ-MGB | 28.02 | 0.018889 |
| MS9-PSS1 (R2) | 1 | cox1 | UNKNOWN | FAM | NFQ-MGB | Undetermined | 0.018889 |
| | 2 | cox1 | UNKNOWN | FAM | NFQ-MGB | 30.35 | 0.018889 |
| | 3 | cox1 | UNKNOWN | FAM | NFQ-MGB | 30.19 | 0.018889 |
| MS9-PSS1 (R3) | 1 | cox1 | UNKNOWN | FAM | NFQ-MGB | Undetermined | 0.018889 |
| | 2 | cox1 | UNKNOWN | FAM | NFQ-MGB | 30.04 | 0.018889 |
| | 3 | cox1 | UNKNOWN | FAM | NFQ-MGB | 29.88 | 0.018889 |
| MS9-ASS2 (R1) | 1 | cox1 | UNKNOWN | FAM | NFQ-MGB | Undetermined | 0.018889 |
| | 2 | cox1 | UNKNOWN | FAM | NFQ-MGB | 28.59 | 0.018889 |
| | 3 | cox1 | UNKNOWN | FAM | NFQ-MGB | 30.61 | 0.018889 |
| MS9-ASS2 (R2) | 1 | cox1 | UNKNOWN | FAM | NFQ-MGB | Undetermined | 0.018889 |
| | 2 | cox1 | UNKNOWN | FAM | NFQ-MGB | 30.00 | 0.018889 |
| | 3 | cox1 | UNKNOWN | FAM | NFQ-MGB | 30.10 | 0.018889 |
| MS9-ASS2 (R3) | 1 | cox1 | UNKNOWN | FAM | NFQ-MGB | 34.0 | 0.018889 |

| | | | | | | | |
|---|---|---|---|---|---|---|---|
| | 2 | cox1 | UNKNOWN | FAM | NFQ-MGB | 30.06 | 0.018889 |
| | 3 | cox1 | UNKNOWN | FAM | NFQ-MGB | 30.09 | 0.018889 |
| MS9-PSS3 (R1) | 1 | cox1 | UNKNOWN | FAM | NFQ-MGB | 33.267 | 0.018889 |
| | 2 | cox1 | UNKNOWN | FAM | NFQ-MGB | 30.36 | 0.018889 |
| | 3 | cox1 | UNKNOWN | FAM | NFQ-MGB | 30.58 | 0.018889 |
| MS9-PSS3 (R2) | 1 | cox1 | UNKNOWN | FAM | NFQ-MGB | 30.95 | 0.018889 |
| | 2 | cox1 | UNKNOWN | FAM | NFQ-MGB | 30.52 | 0.018889 |
| | 3 | cox1 | UNKNOWN | FAM | NFQ-MGB | 30.47 | 0.018889 |
| MS9-PSS3 (R3) | 1 | cox1 | UNKNOWN | FAM | NFQ-MGB | 30.10 | 0.018889 |
| | 2 | cox1 | UNKNOWN | FAM | NFQ-MGB | 30.15 | 0.018889 |
| | 3 | cox1 | UNKNOWN | FAM | NFQ-MGB | Undetermined | 0.018889 |
| Positive | 1 | cox1 | UNKNOWN | FAM | NFQ-MGB | 14.91 | 0.018889 |
| | 2 | cox2 | UNKNOWN | FAM | NFQ-MGB | 14.82 | 0.018889 |
| | 3 | cox3 | UNKNOWN | FAM | NFQ-MGB | 38.77 | 0.018889 |

**Table 3.** Edaphic factors of all soil samples in Barangay Tapel and Magrafil.

| **Sample.** | **Temperature (℃)** | **pH** | **O.M. % (N)** | **P (ppm)** | **K (ppm)** | **Zn (ppm)** | **Cu (ppm)** | **Mn (ppm)** | **Fe (ppm)** | **Ca (cmol/kg)** | **OC (%)** | **OM (%)** |
|---|---|---|---|---|---|---|---|---|---|---|---|---|
| Non-endemic area | 25.0 | 5.74 | 2.13 | 9.40 | 290 | 0.26 | 1.02 | 15.40 | 20.80 | 22.8 | 1.95 | 3.36 |
| TS1-PSS1 | 23.0 | 5.90 | 4.37 | 45.80 | 420 | 9.08 | 5.70 | 89.40 | 239.60 | 7.99 | 2.75 | 4.72 |
| TS1-ASS2 | 23.0 | 5.38 | 4.82 | 42.90 | 400 | 5.18 | 3.00 | 49.20 | 228.80 | 5.61 | 2.77 | 4.77 |

| | | | | | | | | | | | | |
|---|---|---|---|---|---|---|---|---|---|---|---|---|
| TS1-PSS3 | 22.0 | 5.83 | 4.82 | 32.80 | 210 | 4.44 | 2.00 | 35.60 | 237.40 | 6.77 | 2.75 | 4.72 |
| TS2-PSS1 | 24.0 | 5.78 | 4.78 | 42.70 | 325 | 8.30 | 5.28 | 78.60 | 253.80 | 3.72 | 2.93 | 5.04 |
| TS2-ASS2 | 23.0 | 5.22 | 4.92 | 33.20 | 85 | 3.54 | 3.02 | 17.00 | 258.60 | 5.56 | 2.60 | 4.48 |
| TS2-PSS3 | 23.0 | 5.30 | 5.09 | 32.50 | 120 | 3.08 | 1.96 | 24.00 | 228.80 | 7.61 | 3.11 | 5.35 |
| TS3-PSS1 | 23.0 | 5.30 | 5.23 | 31.10 | 95 | 5.96 | 3.46 | 34.00 | 260.40 | 7.04 | 2.86 | 4.93 |
| TS3-ASS2 | 23.5 | 5.20 | 4.88 | 37.90 | 160 | 4.88 | 2.40 | 45.80 | 238.60 | 4.67 | 2.85 | 4.89 |
| TS3-PSS3 | 22.0 | 5.57 | 2.89 | 106.00 | 55 | 7.32 | 11.28 | 130.20 | 114.20 | 10.25 | 2.55 | 4.39 |
| TS4-PSS1 | 23.0 | 5.51 | 3.27 | 13.30 | 280 | 1.64 | 4.38 | 84.50 | 206.15 | 4.84 | 1.87 | 3.22 |
| TS4-ASS2 | 23.0 | 5.34 | 1.96 | 23.90 | 210 | 1.76 | 6.08 | 47.80 | 148.20 | 2.88 | 1.05 | 1.81 |
| TS4-PSS3 | 23.0 | 5.32 | 1.58 | 5.90 | 135 | 1.60 | 4.72 | 66.80 | 140.80 | 2.79 | 0.94 | 1.62 |
| TS5-PSS1 | 22.0 | 5.45 | 2.17 | 12.10 | 160 | 1.96 | 6.04 | 106.80 | 126.00 | 3.05 | 1.27 | 2.19 |
| TS5-ASS2 | 23.0 | 5.43 | 2.48 | 6.80 | 110 | 1.64 | 4.18 | 80.80 | 154.20 | 2.64 | 1.23 | 2.11 |
| TS5-PSS3 | 22.0 | 5.38 | 1.58 | 10.80 | 125 | 1.48 | 4.44 | 81.80 | 142.80 | 2.92 | .91 | 1.56 |
| MS6-PSS1 | 22.0 | 5.54 | 3.34 | 58.30 | 550 | 10.40 | 13.46 | 114.80 | 97.80 | 6.27 | 2.35 | 4.04 |
| MS6-ASS2 | 23.0 | 5.54 | 4.02 | 54.00 | 380 | 13.44 | 16.08 | 161.40 | 121.80 | 0.92 | 2.29 | 3.94 |
| MS6-PSS3 | 23.0 | 5.93 | 3.06 | 64.00 | 780 | 13.48 | 13.86 | 101.00 | 93.00 | 5.63 | 1.62 | 2.78 |
| MS7-PSS1 | 22.0 | 6.07 | 2.54 | 63.70 | 480 | 9.23 | 15.47 | 113.50 | 124.00 | 2.08 | 2.35 | 4.04 |

| | | | | | | | | | | | | |
|---|---|---|---|---|---|---|---|---|---|---|---|---|
| MS7-ASS2 | 23.0 | 5.70 | 2.51 | 54.50 | 430 | 7.74 | 11.28 | 112.80 | 127.60 | 2.19 | 2.12 | 3.64 |
| MS7-PSS3 | 23.0 | 5.95 | 2.72 | 67.00 | 750 | 9.06 | 11.08 | 98.80 | 139.00 | 6.33 | 2.42 | 4.16 |
| MS8-PSS2 | 23.0 | 6.05 | 3.37 | 51.40 | 470 | 9.08 | 11.72 | 97.40 | 82.80 | 4.57 | 1.92 | 3.31 |
| MS8-ASS2 | 23.0 | 6.04 | 3.20 | 38.50 | 530 | 11.08 | 14.04 | 102.60 | 59.60 | 6.07 | 1.89 | 3.26 |
| MS8-PSS3 | 23.0 | 6.09 | 2.54 | 45.70 | 750 | 10.16 | 11.82 | 84.40 | 49.60 | 0.25 | 1.69 | 2.91 |
| MS9-PSS1 | 23.0 | 5.96 | 2.68 | 65.00 | 670 | 10.06 | 14.60 | 134.80 | 80.00 | 2.71 | 1.39 | 2.39 |
| MS9-ASS2 | 23.0 | 6.14 | 3.10 | 45.80 | 400 | 10.52 | 13.50 | 134.80 | 110.00 | 4.47 | 2.16 | 3.72 |
| MS9-PSS3 | 22.0 | 5.99 | 2.75 | 36.90 | 325 | 7.54 | 12.88 | 74.20 | 74.60 | 4.51 | 1.48 | 2.55 |

A

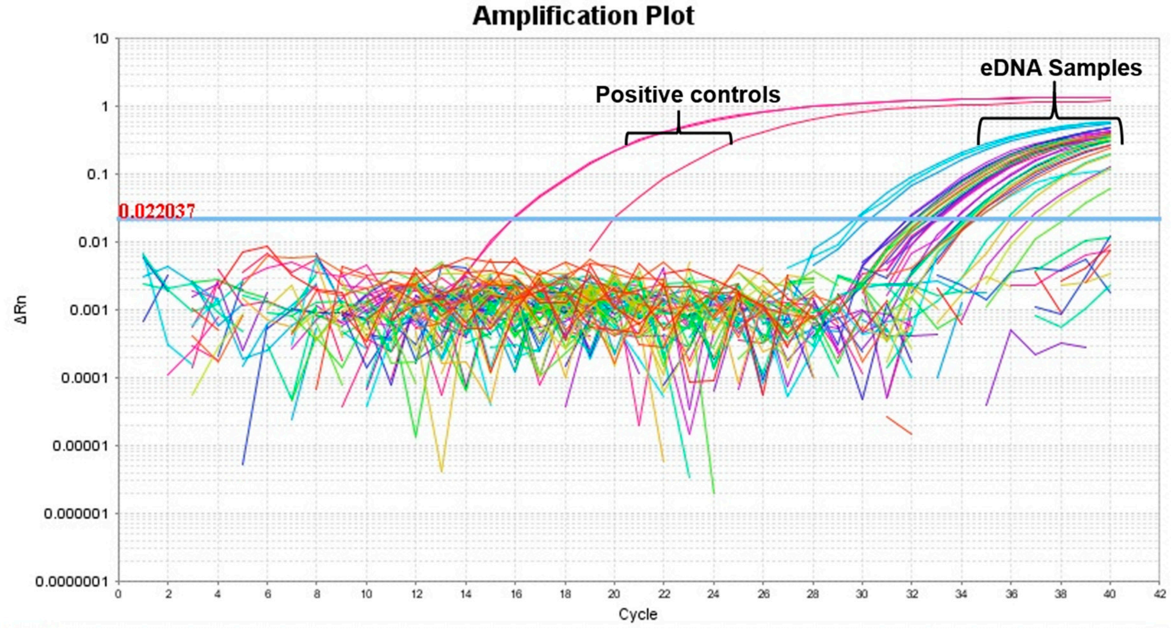
Amplification Plot
Positive controls
eDNA Samples
0.022037
ΔRn
Cycle

B

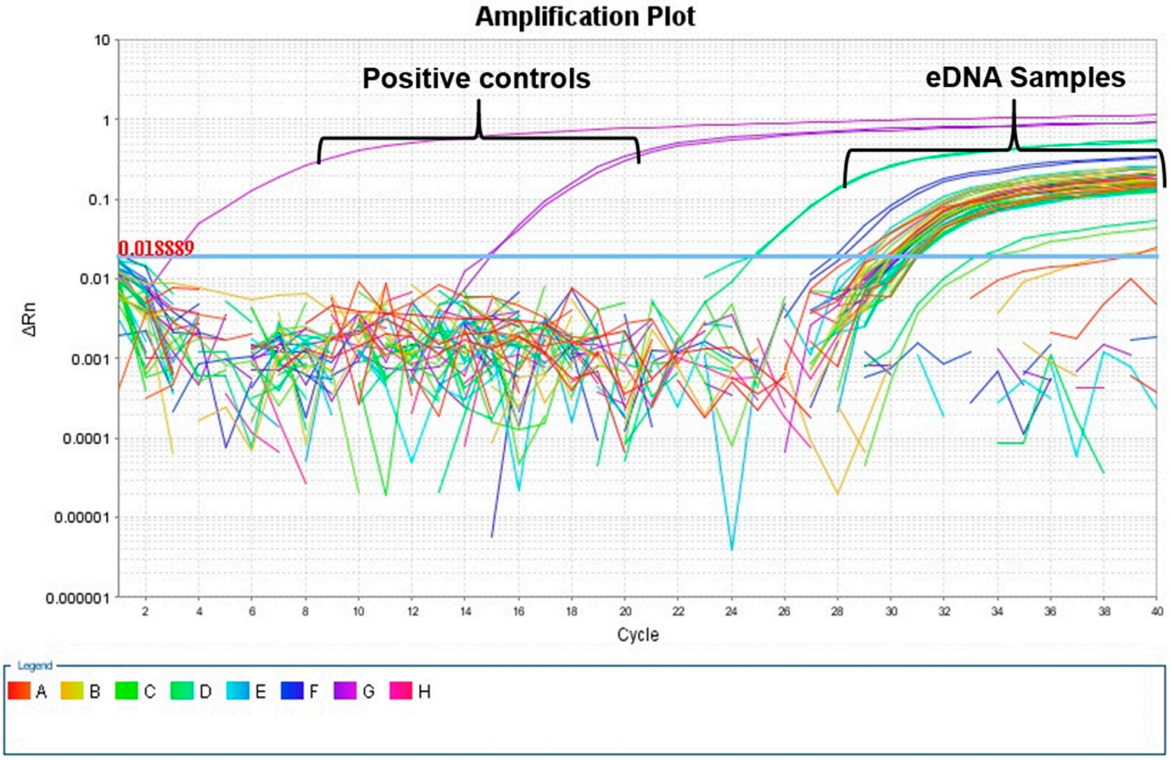
Amplification Plot
Positive controls
eDNA Samples
0.018889
ΔRn
Cycle
Legend
A
B
C
D
E
F
G
H

C

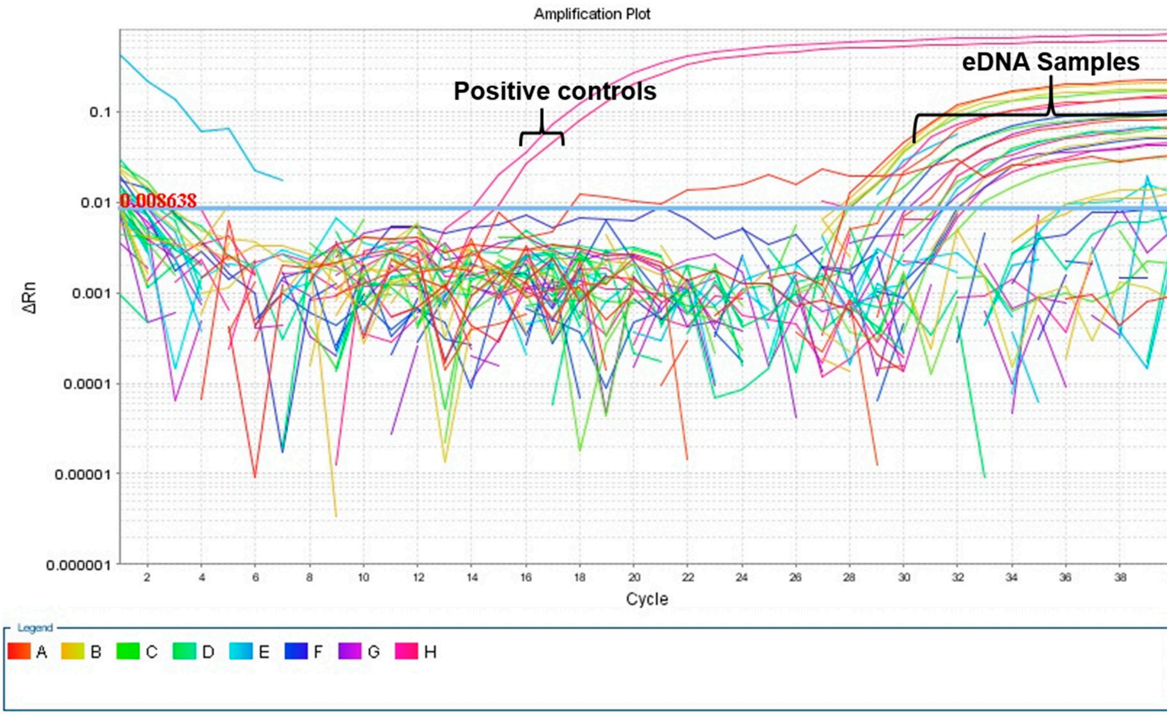


D

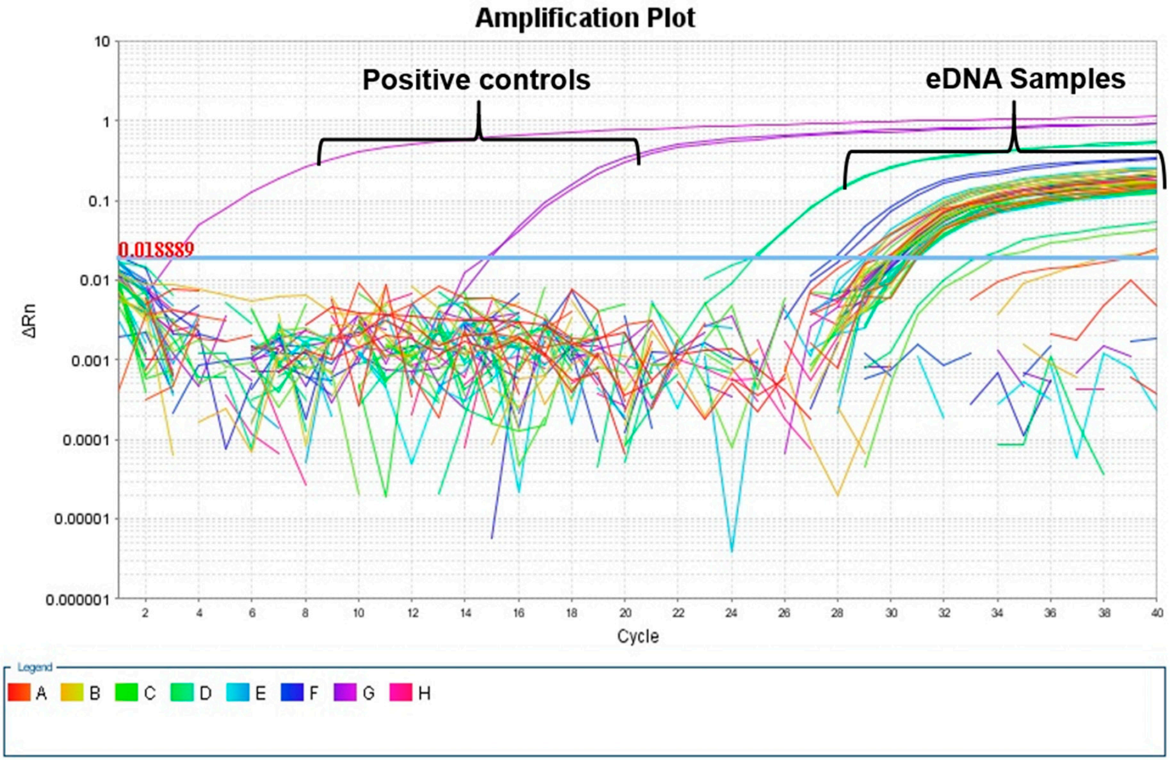


**Figure S4.** TaqMan qPCR amplification plots of *O. hupensis quadrasi*, showing cycle threshold (Ct) levels as indicated by the blue horizontal line; the positive control is indicated by the pink line. The positive result is indicated by amplifications exceeding the threshold line. Each color represents a single sample.
